# Search for chromosomal instability aiding variants reveal naturally occurring kinetochore gene variants that perturb chromosome segregation

**DOI:** 10.1016/j.isci.2024.109007

**Published:** 2024-01-26

**Authors:** Asifa Islam, Janeth Catalina Manjarrez-González, Xinhong Song, Trupti Gore, Viji M. Draviam

**Affiliations:** 1 School of Biological and Chemical Sciences, Queen Mary, University of London, London E1 4NS, UK; 2 London Interdisciplinary Doctoral Program, University College London, London, UK

**Keywords:** Techniques in genetics, Molecular genetics, Phenotyping, Genotyping, Chromosome organization, Cell biology

## Abstract

Chromosomal instability (CIN) is a hallmark of cancers, and CIN-promoting mutations are not fully understood. Here, we report 141 chromosomal instability aiding variant (CIVa) candidates by assessing the prevalence of loss-of-function (LoF) variants in 135 chromosome segregation genes from over 150,000 humans. Unexpectedly, we observe both heterozygous and homozygous CIVa in Astrin and SKA3, two evolutionarily conserved kinetochore and microtubule-associated proteins essential for chromosome segregation. To stratify harmful versus harmless variants, we combine live-cell microscopy and controlled protein expression. We find the naturally occurring Astrin p.Q1012∗ variant is harmful as it fails to localize normally and induces chromosome misalignment and missegregation, in a dominant negative manner. In contrast, the Astrin p.L7Qfs∗21 variant generates a shorter isoform that localizes and functions normally, and the SKA3 p.Q70Kfs∗7 variant allows wild-type SKA complex localisation and function, revealing distinct resilience mechanisms that render these variants harmless. Thus, we present a scalable framework to predict and stratify naturally occurring CIVa, and provide insight into resilience mechanisms that compensate for naturally occurring CIVa.

## Highlights

• We report 141 Chromosomal Instability aiding Variant (CIVa) candidates in humans
• Astrin p.Q1012∗ is harmful; it dominant-negatively aids chromosome missegregation
• Astrin p.L7Qfs∗21 is harmless, expressing a short isoform that localizes normally
• SKA3 p.Q70Kfs∗7 allows endogenous SKA complex’s normal mitotic function

## Graphical abstract

**Figure:**
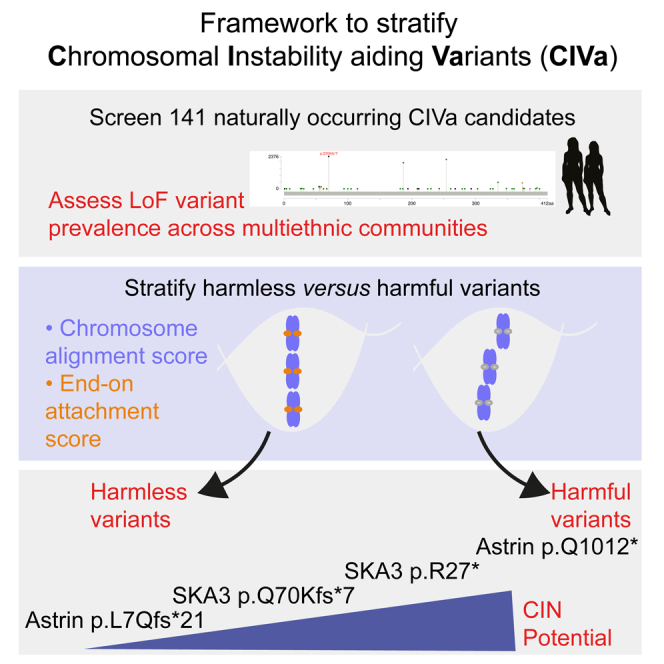

## Introduction

Chromosomal instability (CIN) is a hallmark of several pathologies, including cancers.^1^ CIN causes a high frequency of chromosomal abnormalities and can lead to aneuploidy, with CIN being more dynamic and difficult to quantify compared to aneuploidy.^2^ CIN can arise from errors in the process of chromosome-microtubule attachment, leading to chromosome missegregation, disorganized nucleus, transcriptional heterogeneity, and replication and proteotoxic stress.^3^^,^^4^^,^^5^^,^^6^ Chromosome-microtubule attachment is facilitated by a macromolecular protein structure, the kinetochore (reviewed in Conti et al., and Hara et al.,^7^^,^^8^). Kinetochore-microtubule bridging proteins can be present at kinetochores either throughout mitosis (Ndc80 complex^9^^,^^10^^,^^11^) or recruited via Ndc80 during early mitosis (Spindle and Kinetochore-associated (SKA) complex^12^^,^^13^) in a microtubule-independent manner^14^^,^^15^ or a microtubule-end dependent manner (Astrin-SKAP (Small Kinetochore Associated Protein) complex^16^^,^^17^^,^^18^^,^^19^). Variants in kinetochore genes can cause primary microcephaly (MCPH, OMIM: 604321, OMIM: 616051)^20^ and mosaic variegated aneuploidy (MVA, OMIM: 257300)^21^ that are strongly linked to cancers. Yet, there is no systematic study of chromosome segregation gene variants seen in healthy humans across ancestries.

Genetic disorders are common in communities with consanguineous marriages.^22^^,^^23^^,^^24^ Genetic variants as single copies (heterozygous/monoallelic) can reduce corresponding protein levels.^20^^,^^25^^,^^26^^,^^27^^,^^28^^,^^29^ However, an increase in parental relatedness can increase the chances of biallelic variants leading to a full loss of protein. So, a list of chromosomal instability-aiding variants (CIVa) tolerated in healthy humans in the heterozygous but not homozygous form is critical for precision medicine. Also, information on highly prevalent CIVa candidates in healthy populations are valuable tools to probe how a predicted loss of function (LoF) variant in a chromosome segregation gene is tolerated in cells or specific tissues, expanding our knowledge of chromosome segregation mechanisms and in turn, CIN preventing pathways.

The Genes and Health (GH) database encompasses data from 100,000 individuals of Pakistani and Bangladeshi origin with a high incidence of consanguineal marriages. Genetic databases such as the Genome Aggregation database gnomAD (a coalition of several international population-specific and disease-specific databases^30^) and somatic mutation databases such as COSMIC (database of cancer patients organized by tissue types^31^) are powerful datasets for predicting harmful LoF variants in kinetochore genes. However, no scalable framework to identify and rank the impact of harmful CIVa has been proposed.

Here, we present a scalable and quantitative framework to predict and rank the impact of naturally occurring CIVa. A bioinformatic screen for CIVa candidates in chromosome segregation genes using healthy human genomes led us to several LoF variants in kinetochore and microtubule-binding proteins that form large complexes, the Astrin, SKA, and Ndc80 complexes. By combining live-cell imaging and variant protein expression, we demonstrate the adverse impact of Astrin p.Q1012∗ variant that causes defective localization and chromosome segregation. Unexpectedly, two LoF variants, SKA3 p.Q70Kfs7 and Astrin p.L7Qfs∗21, are tolerated in cells. SKA3 p.Q70Kfs7 may not be incorporated into the larger SKA complex whereas Astrin p.L7Qfs∗21 generates a short isoform that is functional during mitosis. Thus, using live-cell microscopy and variant isoform study, we present a scalable framework to stratify harmful CIVa, and reveal resilience mechanisms that compensate for LoF kinetochore protein variants.

## Results

### Unexpected LoF variants of essential kinetochore genes in healthy humans

**Figure 1:**
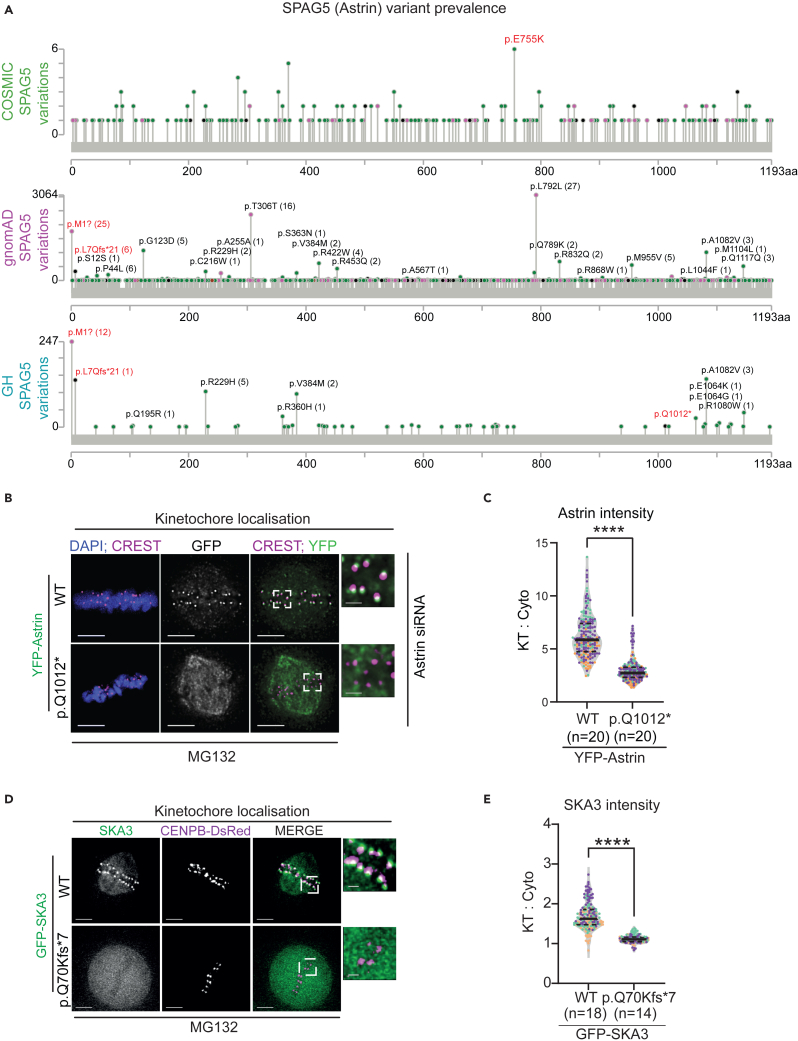
CIVa study reveals LoF kinetochore gene variants in healthy humans (A) Chromosomal instability variant (CIVa) analysis of SPAG5 gene (encoding Astrin) comparing its variant prevalence in COSMIC, gnomAD, and ELGH databases. Images of lollipop graphs show the positions and number of occurrences of different types of variations in the SPAG5 gene, including potential CIVa sites. A black dot indicates a truncating mutation, a green dot indicates a missense mutation and a purple dot indicates other mutation types. The red font indicates key LoF variants. Numbers in brackets indicate the number of homozygous occurrences. (B) Representative immunofluorescence images of Astrin wild type and p.Q1012∗ expressing cells treated as in Figures 1 and S2A and probed for GFP and CREST. DNA was stained with DAPI. Scale bars: 5 μm in uncropped images and 1 μm in insets. (C). Violin plot showing differences in YFP-tagged Astrin or pQ1012∗ intensities at the kinetochores. Ratio of kinetochore and cytoplasmic intensities are shown. Colors represent independent experiments. Mann-Whitney U test was performed for statistical significance. “∗∗∗∗” represents p < 0.0001. (D) Representative deconvolved images of live mitotic cells coexpressing CENPB-DsRed (centromere marker) and N-terminal GFP-tagged SKA3 wild-type or p.Q70Kfs∗7 as indicated (n = 24 wild-type and 15 p.Q70Kfs∗7 mitotic cells). (E) Violin plot showing differences in GFP tagged SKA3 wild-type or p.Q70Kfs∗7 intensities on individual kinetochores, marked using centromeric marker CENPB-DsRed in metaphase arrested cells as indicated in D (n values of cells as indicated). Ratio of kinetochore and cytoplasmic intensities are shown. Circles represent individual kinetochores and colors represent independent experiments. Mann-Whitney U test was performed for statistical significance. “∗∗∗∗” represents p < 0.0001. The black bars represent mean values. Scale bars: 5 microns in uncropped images and 1 micron in insets.

To identify CIVa candidates, we conducted the first bioinformatics screen for predicted LoF variants in 135 chromosome segregation genes using the GH database^32^ (heterozygous and homozygous variants, from multiple databases, are collated in Table S1). To screen for LoF variant candidates, we analyzed the prevalence of premature stop-codons, due to a missense mutation or nucleotide loss/gain associated frameshift, in at least two different individuals (germline variants and allelic prevalence listed in Table S1). Out of the 141 potential CIVa identified, only 6 are found as homozygous (biallelic) variants which include Astrin and SKA3, kinetochore proteins essential for accurate chromosome segregation.^17^^,^^33^^,^^34^^,^^35^ To test whether two LoF variants in the SPAG5/Astrin gene, p.(Q1012∗) and p.(L7Qfs∗21) are specific to the population surveyed in the GH database, we screened wider population databases, COSMIC (cancer database) and gnomAD (population and disease-specific database) (Figure 1A). The Astrin p.(L7Qfs∗21) variant is not found in the COSMIC database, but found in gnomAD as both heterozygous and homozygous forms (Table S1; Figure 1A),^36^ and the variant is exclusive to South Asians (with an allele frequency of 2%) in the 1000 Genomes Project Phase 3.^37^ In contrast, a variant of SKA3, SKA3Q70Kfs∗7 is found in healthy humans,^32^ and also reported in multiple cancers including hematopoietic, lymphoid, thyroid, pancreatic, esophageal and lung^31^ (Figure S1A). In summary, some of the kinetochore gene variants in the GH database can be found in other databases. We present the first queryable resource of CIVa candidates, including their allele frequencies, in 135 human chromosome segregation genes collated across different databases here: https://github.com/Draviam-lab/CIVa.

To test whether Astrin variants are selectively depleted in cancers, we probed the incidence of somatic mutations in Astrin and Ndc80 (an interactor of Astrin)^35^^,^^38^ by comparing multiple tumor tissues for variants in five gene categories: (a) MCPH genes, (b) MVA genes, (c) the Astrin-SKAP complex, (d) Astrin-SKAP interactors, and (e) TP53 and BRCA1 (tumor suppressor genes, as positive controls). As expected, mutations in TP53 were found in all tissue types, including gastrointestinal, placenta, and pleura (Figure S1B). TP53 mutation was above 50% in most tumor types, showing its high prevalence (Figure S1B). In contrast, mutations in BRCA1, MCPH genes and most of the Astrin-SKAP interacting partners, including Ndc80 complex,^35^^,^^38^ are not present in all tissue types suggesting tissue-specificity (Figure S1B). In addition, the frequencies of mutations in the Astrin-SKAP and Ndc80 complexes are much lower compared to MCPH or BRCA1 genes (Figure S1B, see box). Thus, somatic mutations in the Astrin-SKAP, NDC80 complex, and MVA genes are not high, highlighting the uniqueness of the two LoF gene variants uncovered in the CIVa database: Astrin p.(Q1012∗) and p.(L7Qfs∗21).

### The rare variant astrin p.Q1012∗ shows impaired kinetochore localization

We set out to develop an easily scalable framework for quantifying the impact of CIVa candidates by probing their localization and function in spindle checkpoint proficient HeLa transformed epithelial cell line. The Astrin p.(Q1012∗) variant is predicted to eliminate Astrin’s C-terminal tail that is required for the protein’s recruitment to kinetochores.^35^ To assess Astrin p.(Q1012∗) variant localization, we transiently expressed Yellow Fluorescent Protein (YFP)-tagged Astrin wild-type (WT) or p.Q1012∗ in HeLa, both in the presence and absence of endogenous Astrin (Figure S2A). We depleted endogenous Astrin using siRNA and expressed siRNA-resistant YFP-tagged Astrin WT or p.Q1012∗ variant protein (Figure S2B). Immunostaining studies of metaphase-arrested mitotic cells, with equatorially centered spindles,^39^ showed that unlike YFP-Astrin WT protein, the p.Q1012∗ variant fails to localize at kinetochores identified by anti-centromere (CREST) anti-sera (Figures 1B and 1C). Qualitative analysis of Astrin’s enrichment at kinetochores (crescent-like signals) showed that while YFP-Astrin WT localizes on the mitotic spindle and kinetochores (as crescents), Astrin p.Q1012∗ localizes exclusively on the mitotic spindle but is not enriched on kinetochores (Figures 1B, 1C, and S2C). While the mislocalization of Astrin p.Q1012∗ is most striking in Astrin siRNA treated cells, the kinetochore localization defect can be observed in cells with no siRNA treatment (see in the following section). We compared p.Q1012∗ localization against two previously reported Astrin C-terminal mutants with different extents of kinetochore localization defects^35^ (Figures S2D‒S2F). Quantitative analysis of Astrin crescents at the outer-kinetochores of immunostained cells showed that the kinetochore enrichment of Astrin p.Q1012∗ is impaired similarly to the Astrin Δ70 deletion mutant and more severely compared to the Astrin 4A mutant (Figures S2E and S2F). Together these findings demonstrate Astrin p.Q1012∗ as a variant exhibiting a severe loss of kinetochore localization.

### A high-frequency LoF variant in SKA3 allows chromosome congression

We next explored LoF kinetochore variants observed in high frequency. The SKA3 p.(Q70Kfs∗7) variant (1906 heterozygous; 2 homozygous)^32^ is expected to express a truncated SKA3 which can disrupt the oligomerization domain that brings together the 3 SKA complex subunits (SKA1-SKA2-SKA3)^34^ (Figure S3A). To assess SKA3 variant’s localization during mitosis, we coexpressed either GFP-SKA3 WT or GFP-SKA3 p.Q70Kfs∗7 with CENPB-dsRed (a centromere marker). Live-cell microscopy showed that unlike GFP-SKA3 WT, the GFP-SKA3 p.Q70Kfs∗7 variant fails to localize at the kinetochores and microtubules of the mitotic spindle (Figures 1D and 1E). Immunoblotting studies showed the expression of a truncated SKA3 in cells transfected with plasmids encoding GFP-SKA3 p.Q70Kfs∗7 (Figure S3B). However, no chromosome congression defects were observed in metaphase arrested cells expressing GFP-SKA3 p.Q70Kfs∗7 (17 of 18 cells; Figure 1D), suggesting normal kinetochore-microtubule attachments.

**Figure 2:**
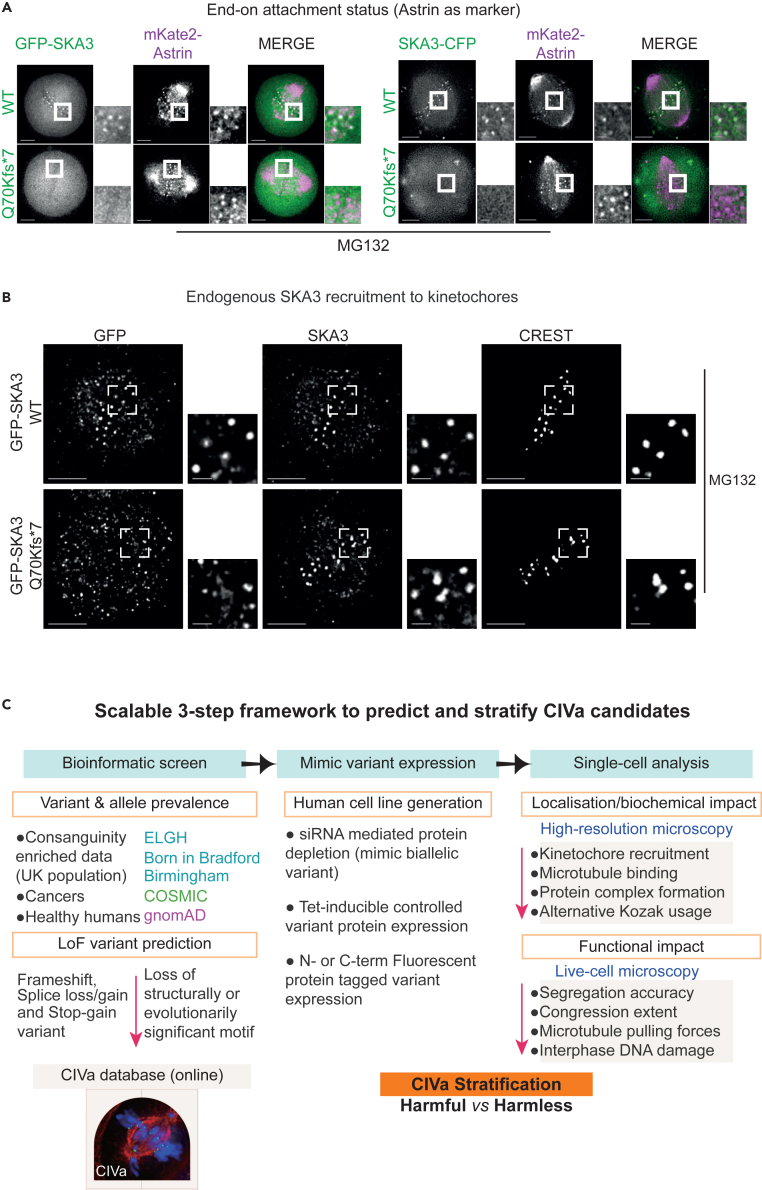
High-frequency SKA3 p.Q70Kfs∗7 does not disrupt end-on chromosome-microtubule attachment (A) Representative deconvolved images of live mitotic cells coexpressing mKate2-Astrin (end-on attachment marker) along with N-terminally tagged GFP-SKA3 wild-type or p.Q70Kfs∗7 or C-terminally tagged SKA3-CFP wild-type or p.Q70Kfs∗7 as indicated (n = 15 wild-type and 12 p.Q70Kfs∗7 mitotic cells). Samples collated from at least 3 independent experimental repeats. Scale bars: 5 μm in uncropped images and 1 μm in insets. (B) Representative immunofluorescence images of GFP tagged SKA3 wild-type or p.Q70Kfs∗7 expressing cells treated with MG132 and probed using anti-GFP and anti-SKA3 antibodies and CREST antisera. Anti-SKA3 antibody recognizes 156–177 a.a which follows the premature stop codon in SKA3 p.Q70Kfs∗7. (C) Cartoon of a three-step scalable framework used to predict, mimic and stratify CIVa on the basis of their impact on kinetochore protein localization and function assessed using microscopy assays. Bioinformatic predictions on LoF variants in kinetochore genes and their allelic prevalence across COSMIC, gnomAD, and ELGH databases are collated into the CIVa database. CIVa in kinetochore genes are mimicked in single cells using different protein expression tools and their impact is quantitatively assessed to stratify harmful and harmless variants in monoallelic and biallelic forms.

To probe whether mature kinetochore attachments can form in variant expressing cells, we used the end-on attachment marker, Astrin-SKAP complex.^7^ Live-cells coexpressing mKate2-Astrin showed normal Astrin localization in cell expressing SKA3-WT or Q70Kfs∗7 tagged at the C or N-termini, demonstrating mature end-on attachments and confirming the lack of variant localization at kinetochores (Figure 2A). Immunostaining using antibodies against 156–177 a.a of SKA3 (Figure S3A) showed that in cells expressing GFP-SKA3 p.Q70Kfs∗7, endogenous SKA3 localizes normally at kinetochores marked with CREST antisera (Figure 2B), which suggest normal endogenous SKA complex formation. In summary, the SKA3 Q70Kfs∗7 variant leads to a truncated SKA3 that does not localize at kinetochores and does not interfere with chromosome congression or Astrin recruitment, indicating normal chromosome-microtubule attachments in cells expressing SKA3 p.(Q70Kfs∗7).

Another high-frequency SKA3 p.(R27∗) variant was found in COSMIC and gnomAD (6805 heterozygous; 0 homozygous; gnomAD) but not in the GH database. Homozygous variants of SKA3 p.(R27∗) do not exist despite the prevalence of the heterozygous form, relative to other variants in the same gene (Figure S4A). Similar to SKA3 p.(Q70Kfs∗7), SKA3 p.R27∗ expression did not affect chromosome congression but resulted in the expression of a short fragment that is expected to include the dimerization domain of SKA3 (Figures S3, S4B, and S4C). We conclude SKA3 p.(Q70Kfs∗7) and p.(R27∗) may be generally harmless in the presence of full-length SKA3, explaining their monoallelic prevalence across ancestries. Thus, our 3-step framework to predict CIVa candidates, mimic their expression, and analyze their impact using single-cell studies can help stratify harmless versus harmful variants in chromosome segregation genes (Figure 2C).

### High-frequency astrin p.(L7Qfs∗21) variant reveals alternative kozak usage as a resilience mechanism

**Figure 3:**
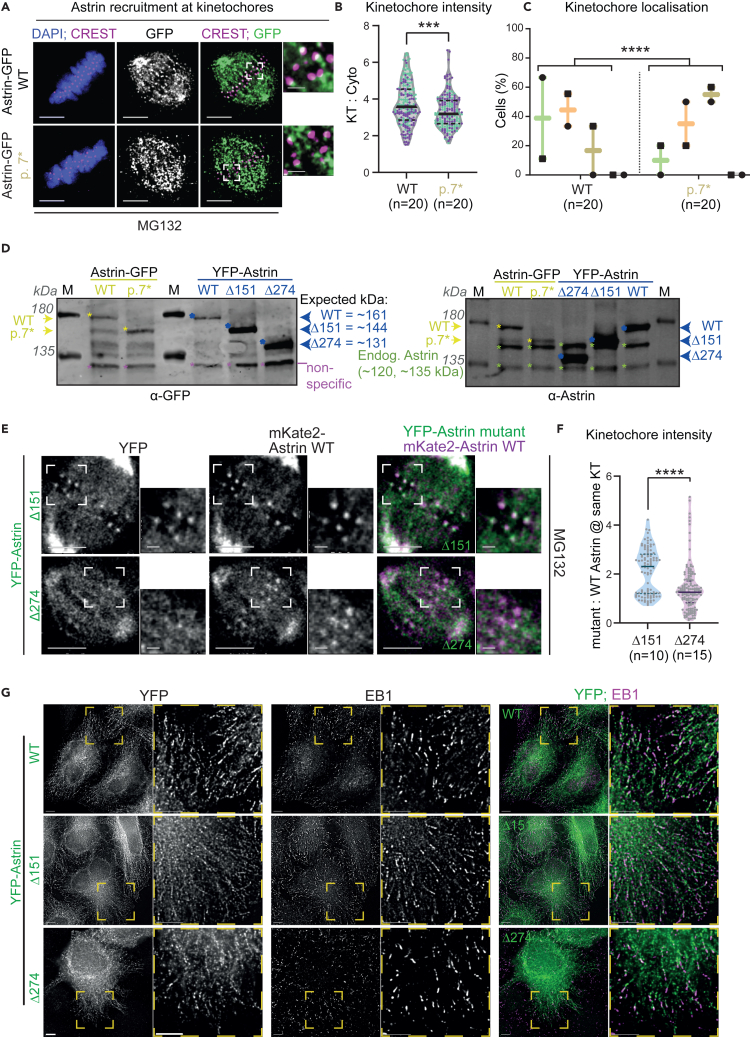
Short Astrin isoform localizes on microtubules and at kinetochores (A) Representative immunofluorescence images of Astrin wild type and Astrin p.7∗ expressing cells treated with MG132 and immunostained using anti-GFP antibody and CREST antisera (as a kinetochore marker). DNA was stained with DAPI. Scale bars: 5 μm in uncropped images and 1 μm in insets. (B) Violin plot showing YFP-Astrin intensity at the kinetochores. The solid line is the median and the dotted lines are quartiles. Circles represent individual kinetochores and colors represent different sets. Mann-Whitney U test was performed for statistical significance. “∗∗∗” represents p < 0.001. (C) Boxplot showing Astrin localization at the kinetochores scored as high, medium, low and no crescents (as in Figure S2A). Symbols represent independent experiments. A chi-square test was performed for statistical significance. “∗∗∗∗” represents p < 0.0001. (D) Immunoblots of HeLa cell lysates expressing Astrin-GFP (wild type and p.7∗) and YFP-Astrin (wild type, Δ151 and Δ274) and probed for GFP and Astrin. Yellow and green asterisks refer to bands corresponding to GFP fusion protein and endogenous Astrin, respectively. Purple asterisk refer to non-specific bands in anti-GFP immunoblot. (E) Representative deconvolved images of live-cells co-expressing YFP-tagged Astrin wild type and either Δ151 or Δ274 Astrin mutant following MG132 treatment. Scale bars: 5 μm in uncropped images and 1 μm in insets. (F) Violin plot showing the ratio of Δ151 or Δ274 Astrin mutant and Astrin wild-type intensities at kinetochores. The solid line is the median and the dotted lines are quartiles. Circles represent individual kinetochores. Mann-Whitney U test was performed for statistical significance. “∗∗∗∗” represents p < 0.0001. (G) Representative immunofluorescence images of YFP-tagged Astrin wild type, Δ151 or Δ274 expressing cells immunostained using antibodies against GFP (for YFP tag) and the growing microtubule-end marker, EB1 that associates with Astrin-SKAP complex.^40^^,^^41^ Scale bars: 5 μm in uncropped images and 5 μm in insets.

To understand the impact of Astrin p.(L7Qfs∗21) variant, we probed its localization using a C-terminal YFP-tagged Astrin with a stop codon at 7 a.a (Figure S5A). Immunostaining showed that C-terminal YFP-tagged Astrin WT (positive control) and 7∗ mutant localized along spindle microtubules (Figure 3A). As reported,^35^ fusing GFP to Astrin’s C-terminus reduces its kinetochore enrichment (Figure 3A). Similar to WT, Astrin 7∗-GFP was reduced at kinetochores (Figures 3A–3C), suggesting a shorter isoform of Astrin may be recruited to the kinetochore.

Screening for alternative translation start sites in Astrin p.(L7Qfs∗21) using Kozak consensus sequences ((gcc)gccRccAUGG) predicted two sites, N-454 and N-823, which precede the region encoding coiled coil stretches in Astrin. To mimic these start sites, we generated two Astrin N-terminal deletion mutants, Δ151 and Δ274 tagged with YFP at their N-termini (Figure S5B). Comparing their corresponding protein sizes using immunoblotting showed that the two N-terminally tagged Astrin deletion mutants, Δ151 and Δ274, migrated at 144 kDa and 131 kDa, respectively (Figure 3D, higher than endogenous Astrin as expected). Importantly, YFP-Astrin Δ151 migrated similarly to Astrin p.L7∗-GFP, in anti-Astrin and anti-GFP antibody-stained immunoblots, indicating that Astrin p.(L7Qfs∗21) can promote the expression of an N-terminally truncated protein starting from 152 a.a. of Astrin.

While Astrin Δ151 mutant localizes normally at the kinetochore, Δ274 mutant exhibits reduced kinetochore localization, compared to mKate2-Astrin that was either coexpressed in live-cells (Figures 3E and 3F) or separately expressed as YFP-Astrin WT control in fixed-cell studies (Figures S6A‒S6C). However, in the absence of endogenous Astrin, Δ274 mutant localizes normally at the kinetochore similar to Astrin WT (Figures S6D, S6E, 2D, and 2E) and it allows the recruitment of SKAP (a member of the Astrin-SKAP complex^38^) that requires Astrin for its localization at kinetochores.^17^ Thus in the absence of full-length protein, shorter isoforms of Astrin can localize at the kinetochore, and recruit SKAP normally.

During interphase, both Astrin Δ274 and Astrin Δ151 are normally excluded from the nucleus and present at growing microtubule ends marked by EB1 an interactor of Astrin complex,^17^^,^^40^ suggesting normal interphase localization and function (Figure 3G). We conclude that Astrin p.(L7Qfs∗21) variant expresses a shorter isoform lacking the first 151 a.a of Astrin, which localizes normally at microtubule-ends, the mitotic spindle and kinetochores. Thus, the Astrin p.(L7Qfs∗21) variant reveals alternative Kozak usage as a resilience mechanism allowing normal chromosome-microtubule attachments.

### Astrin pQ1012∗ abrogates endogenous Astrin-SKAP localization and microtubule-mediated pulling of chromosomes

Of the variants we analyzed, Astrin p.(Q1012∗) is unique with impaired kinetochore localization (Figure 1B) even in the presence of endogenous Astrin. As the Astrin-SKAP complex is a dimer,^38^ we hypothesized that Astrin p.Q1012∗ may disrupt the localization of the endogenous Astrin-SKAP complex. To test this, we transiently expressed the Astrin WT and the p.Q1012∗ variant in HeLa cells and analyzed the kinetochore localization of endogenous Astrin and SKAP (Figure S7A). Immunostaining showed that Astrin localizes as a crescent at an average of 68% of kinetochores in Astrin p.Q1012∗ expressing cells compared to 96% of kinetochores in Astrin WT expressing cells (Figures S7B and S7C). Similarly, endogenous SKAP localizes at ∼86% of kinetochores in Astrin p.Q1012∗ expressing cells compared to 97% of kinetochores in WT expressing cells (Figures S7D and S7E). These findings indicate that the expression of Astrin p.Q1012∗ can disrupt the kinetochore localization of the endogenous Astrin-SKAP complex in a dominant-negative manner.

Astrin’s C-terminal tail, lost in Astrin p.Q1012∗, serves two important roles: it delivers PP1 phosphatase to the kinetochore which stabilizes end-on kinetochore-microtubule attachments and it enables microtubule-mediated pulling which ensures maximum enrichment of Astrin-SKAP at kinetochores.^19^^,^^35^^,^^42^ So, we investigated the extent to which microtubule-mediated pulling and Astrin p.Q1012∗ localization is reduced following Astrin p.Q1012∗ expression. For this, we measured inter-centromeric distances by co-expressing CENPB-DsRed, a centromere marker, with YFP-tagged Astrin WT or p.Q1012∗ (Figures S8A and S8B). The range of inter-centromere distances was reduced in cells expressing YFP-Astrin p.Q1012∗ compared to YFP-Astrin WT (Figures S8B‒S8D, Videos S1 and S2). This difference in inter-centromere distances was more striking following the normalization of inter-centromere distances of each pair to its unstretched state (marked T_0_), indicating sustained reduction in pulling forces. Tracking the fate of sister kinetochores for 5 min showed that although centromeric stretching can be observed in Astrin p.Q1012∗ expressing cells, the maximum inter-centromeric distances are reduced compared to Astrin WT expressing cells (Figures S8E and S8F). Thus, stable microtubule-mediated pulling of kinetochores is reduced following Astrin pQ1012∗ variant expression.

**Figure 4:**
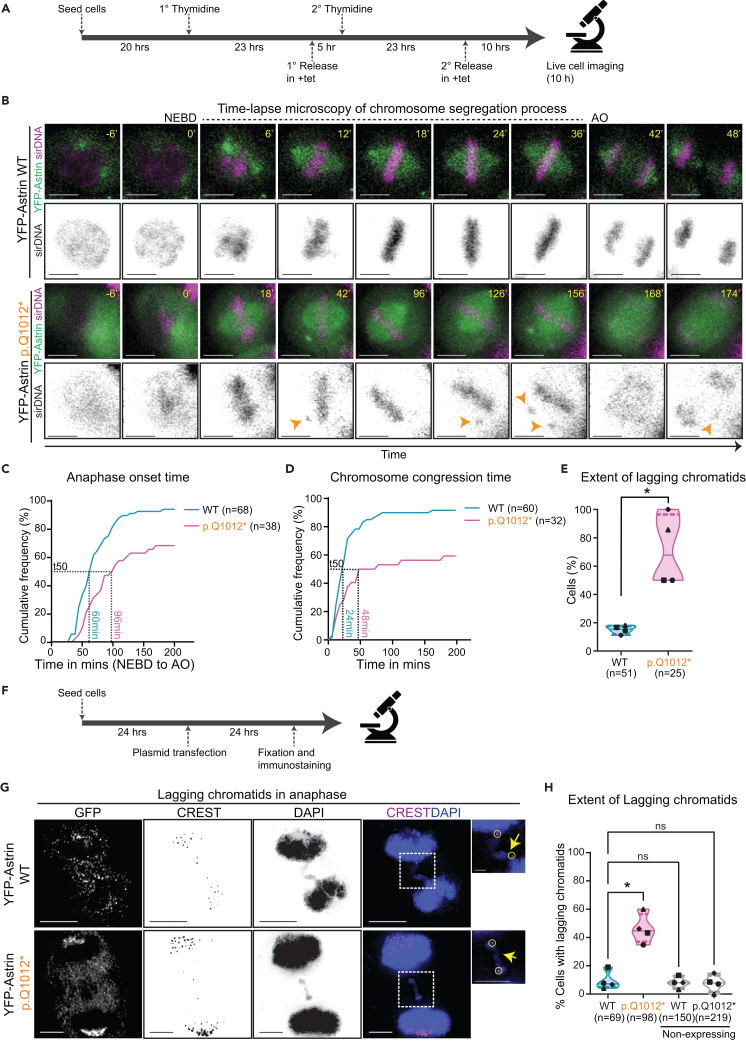
Astrin p.Q1012∗ prolongs mitosis and increases the incidence of lagging chromosomes (A) Experimental regimen showing methodology for double thymidine-based cell cycle synchronization and controlled Tet inducible expression and imaging of Astrin wild-type or variant expressing cells. (B) Representative time-lapse images of Astrin wild type and p.Q1012∗ cells treated as in A. Arrows mark chromosomes that fail to remain congressed and anaphase lagging chromatids. NEBD is nuclear envelope breakdown and AO is anaphase onset. Scale bars: 15 μm. (C) Cumulative frequency graph showing the time taken from NEBD to AO. T50 indicates the time taken by 50% of cells to complete mitosis. (D) Cumulative frequency graph showing the time taken from nuclear envelope break down (NEBD) to the formation of the metaphase plate. T50 indicates the time taken by 50% of cells to congress chromosomes. (E) Violin plot showing the percentage of cells with lagging chromosomes at anaphase. The solid line represents the median and the dotted lines represent the quartiles. Each dot represents an independent set. Paired t-test was performed to find statistical significance. “∗” represents p < 0.05. (C–E) Data represent four independent sets. (F) Experimental regimen (G). Representative immunofluorescence images of Astrin wild type and p.Q1012∗ expressing anaphase cells with lagging chromosomes. Cells were treated as in F and probed for GFP and CREST. DNA was stained with DAPI. Scale bars: 5 μm in uncropped images and 1 μm in insets. (H) Violin plot showing the percentage of anaphase cells with lagging chromosomes. The solid line represents the median and the dotted lines represent quartiles. Each dot represents an independent experiment. One-way ANOVA with DUNNET correction was performed to find statistical significance. “∗” and ns represent p < 0.05 and “not significant”, respectively.

Video S1. Includes time-lapse images of YFP Astrin-WT expressing cell shown in Figure S8 C (Upper row), related to Figure 4

Video S2. Includes time-lapse images of YFP Astrin-Q1012∗ variant expressing cell shown in Figure S8 C (Lower row), related to Figure 4

Quantifying the dynamic loss of kinetochore-bound Astrin p.Q1012∗ relative to WT in metaphase kinetochores through time required automated analysis. We developed a computational workflow using segmentation and particle tracking tools (see STAR Methods) to apply CENPB-DsRed intensities as a mask, to measure YFP-Astrin p.Q1012∗ and WT protein intensities in dynamically stretching sister kinetochores (Figure S9A). While Astrin WT signals at kinetochore were on average 1.1– to 1.3-fold higher than signal intensities in the cytoplasm, the variant was 0.9-fold relative to cytoplasmic signal intensities, indicating a reduction of Astrin p.Q1012∗ at the kinetochore (Figure S9B). Tracking changes through time showed a steady reduction in p.Q1012∗ associated kinetochore intensities (Figure S9C). Thus, the severe reduction of Astrin p.Q1012∗ variant levels at kinetochores correlates well with the sustained reduction in microtubule-mediated pulling across sister kinetochores.

### Astrin p.Q1012∗ expressing cells display prolonged mitosis

The kinetochore localization defect of Astrin p.Q1012∗ is similar to that observed in a C-terminal deletion mutant, Astrin Δ70, which impedes chromosome segregation.^35^ So we investigated the fate of mitotic cells expressing YFP-tagged Astrin p.Q1012∗ by generating a tetracycline-inducible HeLa FRT/TO^TM^ YFP-Astrin Q1012∗ cell line and acquired time-lapse images every 6 min for 10 h in the presence of SiR-DNA (a DNA tracker) following a brief exposure to tetracycline^43^ (Figure 4A). On average 70% of Astrin p.Q1012∗ expressing cells completed mitosis compared to 95% of Astrin WT expressing cells (Figure S10A). Astrin p.Q1012∗ expressing cells that completed mitosis displayed anaphase onset (AO) delay. The time from nuclear envelope break down (NEBD) to AO was 1.5-fold longer in YFP-Astrin p.Q1012∗ expressing cells compared to YFP-Astrin WT expressing cells (96 min for p.Q1012∗ versus 60 min for WT), showing significant delay in AO (Figures 4B and 4C). Both the delay in AO and increased incidence of mitotic failure show that the Astrin p.Q1012∗ variant promotes chromosome missegregation.

To investigate the cause for the prolonged mitosis in Astrin p.Q1012∗ expressing cells, we analyzed chromosome congression using time-lapse movies. Only an average of 56% of Astrin p.Q1012∗ expressing cells congressed their chromosomes compared to 90% of Astrin WT expressing cells (Figures 4B and 4D). Additionally, cells expressing Astrin p.Q1012∗ are 2-fold slower in chromosome congression compared to those expressing Astrin WT; (t50 of NEBD to metaphase: 48 min for p.Q1012∗ versus 24 min for WT; Figures 4B and 4D). Importantly, only an average of 30% of Astrin p.Q1012∗ expressing cells, maintain chromosome congression compared to 76% of Astrin WT expressing cells (Figure S10B). Thus, both the establishment and maintenance of congressed chromosomes are disrupted in YFP-Astrin Q1012∗ expressing cells, confirming defects in maintaining stable chromosome-microtubule attachment which can cause chromosome missegregation.

### Astrin p.Q1012∗ expression promotes chromosome missegregation

To investigate whether the prolonged mitosis, reduced microtubule pulling and chromosome congression defects induced by Astrin p.Q1012∗ have an impact on chromosome segregation accuracy, we analyzed the presence of lagging chromosomes in anaphase cells. Time-lapse movies showed that an average of 70% of Astrin p.Q1012∗ expressing cells presented lagging chromosomes during anaphase compared to 15% of Astrin WT expressing cells (Figures 4B and 4E). Moreover, immunostaining studies showed that an average of 46% of Astrin p.Q1012∗ expressing anaphase cells display lagging chromatids compared to 9% in Astrin WT expressing cells (Figures 4F–4H). Thus, Astrin p.Q1012∗ variant significantly increases the incidence of missegregating chromosomes and lagging chromatids during anaphase. We conclude that the expression of the naturally occurring variant Astrin p.(Q1012∗) despite the presence of Astrin full-length protein (as in heterozygous/monoallelic form) is likely to interfere with chromosomal stability in humans. Unlike previously reported Astrin variants (p.[(G1064E∗3)]; [(K409Pfs∗19)]) presenting clinical features (microcephaly) due to compromised centrosomal localization^20^ here, we present the first assessment of Astrin variants’ loss of kinetochore localization and its impact on chromosome segregation.

## Discussion

We present the first comprehensive survey of CIVa in chromosome segregation genes, and their allelic prevalence, exploiting genome sequencing efforts across multi-ethnic populations. Using a 3-step scalable framework, we predict and stratify CIVa candidates in chromosome segregation genes (Figure S11). We identify a rare LoF variant in the microtubule-associated outer-kinetochore protein Astrin p.Q1012∗; this variant is harmful as it impairs the localization and function of endogenous protein in a dominant negative manner (Figure 4B), showcasing it as a CIN aiding variant in heterozygous (monoallelic) form. Second, we report a high-frequency Astrin p.L7Qfs∗21 variant which expresses a shorter Astrin that localizes and functions normally at microtubule-ends and kinetochores, revealing alternate Kozak usage as a resilience mechanism to cope with harmful LoF variants in homozygous and heterozygous forms. Third, we report SKA3 p.Q70Kfs∗7 which does not affect the function of endogenous full-length SKA3 but may be harmful in homozygous form. Thus, the CIVa database and the framework to stratify the impact of CIVa candidates can help shed light on the origins of CIN in a variety of pathologies.

We exploit the HeLa epithelial cell line that has a robust spindle checkpoint allowing a rapid quantitative assessment of subtle congression defects that promote CIN.^44^^,^^45^ Of the three Astrin variants we explore p.Q1012∗ is unique in disrupting chromosome alignment, reducing microtubule-mediated pulling and increasing chromosome missegregation (Figure S11). Unlike previous studies that show the role of Astrin C-terminal tail in preventing chromosome missegregation using conditions lacking endogenous Astrin,^35^^,^^42^ the current study reveals the dominant negative impact of Astrin p.Q1012∗ variant in the presence of endogenous full length Astrin, a condition that closely mimics the naturally occuring monoallelic variant. Whether the p.Q1012∗ variant affects other cells or tissue types will be informative to explore using non-transformed cells from different tissues.

The SKA3 variant p.(Q70Kfs∗7) introduces a premature termination leading to a truncated protein predicted to disrupt the multimerization domain within the SKA complex which can weaken microtubule attachments and disrupt chromosome alignment.^34^^,^^46^^,^^47^ In cells expressing the SKA3 variant p.(Q70Kfs∗7), we find normal alignment of chromosomes and proper localization of endogenous SKA3 at kinetochores, suggesting normal assembly of the endogenous SKA complex. The large GFP tag could interfere with SKA3 variant Q70Kfs∗7 function. Nevertheless, recent genomics and proteomics analysis of UK biobank samples show that of the 691 gene-level signals from protein truncating variants, 99.4% were associated with decreased protein levels.^48^ We propose inability to disrupt the larger SKA complex as an explanation for the high prevalence and tolerance of the SKA3 p.(Q70Kfs∗7) variant in heterozygous form.

We describe an intriguing premature stop codon (nonsense) variant, Astrin p.(L7Qfs∗21) found across multiple ancestries (heterozygous/homozygous individuals: 134/1 GH, 13/0, GenomeAsia 100K and 320/6, gnomAD databases).^30^^,^^31^^,^^32^ In addition, we report a homozygous Astrin start-loss variant (heterozygous/homozygous individuals: 223/12 GH, 1761/25 gnomAD and 21/0 GenomeAsia 100K and UK10K databases),^30^^,^^32^^,^^37^ indicating its presence in multiple ancestries with a higher incidence among Europeans.^30^ Heterozygous Astrin start-loss and p.(L7Qfs∗21) variants are also listed on TOPmed databases.^49^^,^^50^ How are these nonsense variants tolerated ? Our study of p.(L7Qfs∗21) localization and analysis of alternate Kozak usage reveals a mitotically functional short isoform of Astrin exposing new pathways in cells that can compensate for the Astrin start-loss and p.L7Qfs∗21 variants. Our single-cell studies show that the short isoform localizes better in the absence of full length endogenous Astrin, which is in alignment with the biallelic forms of the variant across different ancestries. The loss of Astrin’s N-terminus may be non-pathogenic in humans, revealing resilience pathways that N-terminal premature stop codon variants of coiled-coil proteins may use by relying on shorter isoform expression. Thus, our single-cell studies to assess CIVa protein localization and their mitotic function provide a scalable framework, that can take advantage of Artificial Intelligence guided large-scale image analysis,^51^ to exploit genetic variant prevalence across ancestries and to stratify CIVa relevant to a variety of CIN syndromes.

### Limitations of the study

Here, we explore the impact of CIVa candidates using the HeLa cell line, a transformed basal carcinoma line expressing HPV oncogenes;^52^ it will be insightful to consider these studies in nontransformed cell cultures without HPV gene expression.^41^ The framework developed here uncovered the first CIVa in the Astrin gene; this needs to be expanded to stratify CIVa candidates seen in cancers (e.g.,CENPC^31^ or Histone genes^53^) with varying extent of CIN.

## STAR★Methods

### Key resources table

**Table:** 

REAGENT or RESOURCE	SOURCE	IDENTIFIER
**Antibodies**		

Mouse anti-GFP (clones 7.1 and 13.1)	Roche	Cat # 11814460001; RRID:AB_390913
Rabbit anti-GFP polyclonal	Abcam	Cat # ab290; RRID:AB_2313768
Rabbit anri-Astrin polyclonal	Proteintech	Cat # 14726-1-AP; RRID:AB_2919813
Rabbit anti-SKAP polyclonal	Atlas	Cat # HPA042027; RRID:AB_10797378
Mouse anti-SKA3 monoclonal (H-9)	Santa-Cruz	Cat # sc-390966; RRID: AB_3068336
Rabbit anti-SKA3 polyclonal	Abcam	Cat # ab186003; RRID: AB_3068337
Mouse anti-γ-Tubulin monoclonal	Sigma-Aldrich	Cat # T6793; RRID:AB_477585
Nuf2	Meraldi et al., 2004	VMD #4
Mouse anti-PP1 monoclonal (E-9)	Santa-Cruz	Cat # sc-7482; RRID:AB_628177
CREST antisera	Europa	Cat # FZ90C-CS1058
Mouse anti-EB1 monoclonal	BD Bioscience	Cat # 610534; RRID:AB_397891
Rat anti-alpha Tubulin monoclonal	Abcam	Cat # ab6160; RRID:AB_305328
Mouse anti-beta Actin monoclonal	Santa Cruz Biotechnology, Inc	Cat # sc-47778; RRID:AB_626632
Highly Cross-Adsorbed Donkey (Polyclonal) Anti-Rabbit IgG (H+L) Antibody Conjugated to IRDye 800CW	LiCor	Cat # 926-32213; RRID:AB_621848
Donkey (Polyclonal) Anti-Mouse IgG (H+L) Antibody Conjugated to IRDye 680RD	LiCor	Cat # 926-68072; RRID:AB_10953628
Goat (Polyclonal) Anti-Rat IgG (H+L) Antibody Conjugated to IRDye 680RD	LiCor	Cat # 926-68076; RRID:AB_10956590

**Chemicals, peptides, and recombinant proteins**		

DAPI (4',6-Diamidino-2-Phenylindole, Dihydrochloride)	Life Technologies	Cat #D1306; RRID:AB_2629482
sir-DNA kit	Tebu-Bio	Cat # SC007
Turbofect	Fisher	Cat # R0531
DharmaFECT duo	Dharmacon	Cat # T-2010
Oligofectamine	Invitrogen	Cat # 12252011
MG132	TOCRIS	Cat # 1748; CAS: 133407-82-6
Thymidine	Thermo Scientific Chemicals	Cat # A11493.06
DMEM Dulbecco's Modified Eagle's Media (DMEM) high glucose, pyruvate	Gibco™	Cat # 41966052
Fetal Bovine Serum (FBS)	Gibco™	Cat # 10270106
Fetal bovine serum (FBS) Tetracycline free	BioSera	Cat # FB-1001T/500 - 014BS799
Antibiotics (Penicillin and Streptomycin)	Gibco™	Cat # 15140122
Hygromycin	Invitrogen	Cat #10687010
Leibovitz's L15 medium	Invitrogen	Cat # 11415064
Opti-MEM, Reduced Serum Medium, no phenol red	Gibco™	Cat # 11058021
Dulbecco's Phosphate-Buffered Saline (dPBS), no calcium, no magnesium	Gibco™	Cat # 14190250
Tween20	Sigma Aldrich	Cat # P1379
Albumin bovine / fraction V	ACROS Organics	Cat # 240405000

**Deposited data**		

Raw original images	Mendeley Data	https://doi.org/10.17632/76bpv8zf5n.1
Image segmentation codes	Github	/Draviam-lab/CIVa

**Recombinant DNA**		

Plasmid: YFP-Astrin p.Q1012∗	This work	This work
Plasmid: Astrin-GFP p.L7 ∗	This work	This work
Plasmid: YFP-Astrin Δ151	This work	This work
Plasmid: YFP-Astrin Δ274	This work	This work
Plasmid: YFP-Astrin wild-type	Conti et al.	Ximbio deposit
Plasmid: (pEGFP N1) Astrin-GFP WT Res vector	Conti et al.	Ximbio Cat # 157855
Plasmid: mKate2-Astrin	Song et al.	Ximbio deposit
Plasmid: pcDNA5 FRT/TO YFP-Astrin p.Q1012∗	This work	This work
Plasmid: pECFP-N1-SKA3-wild-type-CFP	This work	This work
Plasmid: pECFP-N1-SKA3 p.Q70Kfs∗7-CFP	This work	This work
Plasmid: CENPB-dsRed	Conti et al., 2019	Ximbio Cat# 157862
Plasmid: pCS2-GFP-Ska3-wild-type	Zhang et al. 2017	N/A
Plasmid: pCS2-GFP-Ska3-R27∗	This work	N/A
Plasmid: pCS2-GFP-Ska3-Q70Kfs∗7	This work	N/A

**Oligonucleotides**		

Astrin VMD 52 oligo (UCCCGACAACUCA CAGAGAAAUU)	Dharmacon	Custom order
Stealth RNAi™ siRNA Negative Control, Med GC	Invitrogen	Cat # 12935300

**Software and algorithms**		

MutationMapper	cBioPortal	RRID: SCR_014555
SoftWoRx™	SoftWoRx software	RRID: SCR_019157
GraphPad Prism 9™	GraphPad Sofware	RRID: SCR_002798
scikit-image	Image Processing Library	N/A
RStudio-GGPlot	Data Visualisation Software	N/A
Anaconda/Jupyter Notebook	Computing Platform	N/A
Fiji/ImageJ	NIH – public domain	RRID: SCR_002285
Adobe Illustrator	Adobe Illustrator (Adobe Systems)	RRID: SCR_010279

**Other**		

ø13 mm round coverslips	VWR	Cat # 631-0150
4-well cover glass chambered dishes	Lab-Tek	Cat # 1064716
VECTORSHIELD antifade mounting medium	Vector Laboratories	Cat # H-1000-10

### Resource availability

#### Lead contact

Requests for further information and reagents should be directed to and will be fulfilled by the Lead Contact, Prof Viji M Draviam (v.draviam@qmul.ac.uk).

#### Materials availability

This study has generated new cell lines which will be made available upon request to the lead contact, Prof Viji M Draviam (v.draviam@qmul.ac.uk).

#### Data and code availability

• Image data reported in this paper has been deposited at Mendeley (Mendeley Data, V1 https://doi.org/10.17632/76bpv8zf5n.1) and is publicly available as of the date of publication. Accession numbers are listed in the key resources table.
• Codes generated in this work have been deposited at Github (https://github.com/Draviam-lab/CIVa) and are publicly available as of the date of publication. Accession numbers are listed in the key resources table.
• Any additional information required to reanalyse the data reported in this paper is available from the lead contact upon request.

### Experiment model and study participant details

HeLa cells (ATCC) were cultured in Dulbecco's Modified Eagle's Media (DMEM) supplemented with 10% FCS and antibiotics (Penicillin and Streptomycin). HeLaFRT/TO YFP-Astrin cell lines were cultured in tetracycline-free DMEM supplemented with 10% FCS and antibiotics. YFP-Astrin p.Q1012∗ and Astrin-GFP p.L7∗expression plasmids were generated by site-directed point mutagenesis. YFP-Astrin Δ151 and Δ274 expression plasmids were generated by amplifying regions 152-1193 a.a. and 275-1193 a.a. respectively and subcloning into a YFP expression plasmid. Similarly, YFP-Astrin wild-type, Astrin-GFP wild-type and mKate2-Astrin expression plasmids were generated through PCR and subcloning full length Astrin cDNA into pEYFP, pEGFP and mKate2 expression vectors (previously described).^35^^,^^42^ pcDNA5 FRT/TO YFP-Astrin p.Q1012∗ expression plasmid was generated by subcloning YFP-Astrin p.Q1012∗ into pcDNA5 FRT/TO plasmid.

HeLa FRT/TO YFP-Astrin p.Q1012∗ cell line was generated by transfection of HeLa Flp-In cells with pCDNA5-FRT/TO-YFP-Astrin p.Q1012∗ expression plasmid followed by a brief Hygromycin selection and sorting for YFP positive cells using FACS. HeLa FRT/TO YFP-Astrin wild-type cell line was generated using transfection of Tet-inducible YFP-Astrin expression vector (previously described^35^). Cell lines were tested for Mycoplasma using DAPI staining and PCR assay. Induction of exogenous YFP-Astrin was performed by exposing the cells to the DMEM medium supplemented with Tetracycline/Doxycycline. A single Cytosine nucleotide (position 208) deletion mutant of SKA3 fused to GFP or CFP to generate GFP-SKA3 p.Q70Kfs∗7 or SKA3 p.Q70Kfs∗7-CFP expression plasmids, respectively. Plasmid sequences were confirmed by DNA sequencing.

### Method details

#### Genome databases and access

To curate data, we have used multiple databases. 1000 Genomes project Phase 3, UK10K database, the GH database (sample size=8,921), The Catalogue of Somatic Mutations in Cancer (COSMIC) database (sample size>37,000) and gnomAD (sample size=141,456) were all assessed on: 05-03-2021. Table S1 was built using the GH LoF variants list from 2018 and the lollipop graph was generated using GH all variants data 2020 (both accessed on 29.01.2020). Lollipop graphs were also generated using data from gnomAD v.2.1.1 and COSMIC databases (assessed on 29.01.2020). Cancer mutational spectra heatmap was generated from data using the COSMIC database (assessed on 29.01.2020). Lollipop graphs were generated using MutationMapper (cBioPortal for Cancer Genomics::MutationMapper, assessed on 29.01.2020). The list of Chromosome segregation genes in Table S1 was identified using the Gene Ontology term ‘Kinetochore’ (GO:0000776). For Table S1, COSMICdatabase (v5, released in November 2021) was used.

#### Plasmids transfections and drug treatments

siRNA transfection was performed using Oligofectamine according to the manufacturer's instructions. To target Astrin mRNA, Astrin VMD 52 oligo (UCCCGACAACUCACAGAGAAAUU) was used. Negative control siRNA (12,935–300) was from Invitrogen. Plasmid transfection was performed using TurboFect (Fisher; R0531) or DharmaFECT duo (Dharmacon; T-2010) according to the manufacturer's instructions. In addition to the standard protocol, after 4 h of incubation, the transfection medium was removed and pre-warmed DMEM was added to each well. In co-transfection studies, eukaryotic expression vectors encoding Astrin and CENPB were used in a 3:1 ratio.

Induction of exogenous YFP-Astrin was performed by exposing the cells to the DMEM medium supplemented with Tetracycline/Doxycycline. For localization and inter-centromeric distance studies, cells were treated with 10 μM MG132 (TOCRIS; 1748) for one hour. For the mitotic progression study, cells were synchronised using 2.5 mM Thymidine (ACROS organics).

#### Immunostaining studies

Cells were cultured on ø13 mm round coverslips (VWR; 631-0150) and fixed with ice-cold methanol for one minute. Following fixation, two quick washes with a PBST wash buffer (1X PBS + 0.1% Tween 20) were performed, followed by two washes of 5 minutes each. Coverslips were incubated with (1X PBS + 0.1% Tween 20 + 1%BSA) for 20 minutes, before staining with primary antibodies overnight at 4°C followed by two washes before incubation with secondary antibodies for 30 minutes at room temperature. Finally, coverslips were washed twice with PBST except before mounting onto glass slides when coverslips were quickly rinsed in distilled water. Cells were stained with antibodies against GFP (Roche; 1181446001; 1:1000), GFP (Abcam; ab290; 1:1000), SKAP (Atlas; HPA042027; 1:800), Astrin (Proteintech;14726–1-AP; 1:1000), SKA3 (Santa Cruz; H-9; 1:500) and CREST antisera (Europa; FZ90C-CS1058; 1:2000). DAPI (Sigma) was used to stain DNA. All antibody dilutions were prepared using the blocking buffer. Images of immunostained cells were acquired using 100X/NA1.4 UPlanSApo oil immersion objective on a DeltaVision Core microscope equipped with CoolSnap HQ Camera (Photometrics). Deconvolution of fixed-cell images was performed using SoftWorx™.

#### Live-cell imaging

For live-cell imaging studies, cells were seeded onto 4-well cover glass chambered dishes (Lab-Tek; 1064716) and transferred to Leibovitz's L15 medium (Invitrogen;11415064) for imaging. For low-resolution live-cell imaging, HeLa FRT/TO YFP-Astrin cells were synchronised using a double thymidine block. 100 nM sirDNA (Tebu-bio; SC007) was added 10 hours before image acquisition to stain for DNA. 3Z-planes, 0.6 μm apart, were acquired using a 40X/0.95 UPlanSApoair objective on an Applied Precision DeltaVision Core microscope equipped with a Cascade2 camera under EM mode. Imaging was performed at 37°C using a full-stage incubation chamber set upto allow normal mitosis progression and microtubule dynamics.

For high-resolution live-cell imaging, cells were transfected with plasmid vectors 24 hours before an hour-long 10 μM MG132 treatment to arrest mitotic cells in metaphase. 3Z-planes, 0.6 μm apart, were acquired using a 100X/1.40 UPlanSApooil immersion objective on an Applied Precision DeltaVision Core microscope equipped with a Cascade2 camera under EM mode. For live-cell CFP imaging, Applied Precision DeltaVision Elite microscope equipped with an EDGE sCMOS_5.5 camera with a 60X oil-immersion objective was used. Imaging was performed at 37°C using a full-stage incubation chamber set up to allow normal mitosis progression and microtubule dynamics. SoftWorx™ distance measurement tool was used to find inter-centromeric distances. Additional analysis was conducted on Microsoft Excel and graphs were plotted using GraphPad Prism 9™.

#### Immunoblotting studies

Immunoblotting was performed on proteins separated on 8% or 12% SDS-PAGE gels by transferring them overnight onto Nitrocellulose membranes. Membranes were incubated in primary antibodies against Astrin (Proteintech; 14726–1-AP;1:3000), γ-Tubulin (Sigma-Aldrich; T6793; 1:800), GFP (Abcam; ab290; 1:1000) and SKAP (Atlas; HPA042027; 1:1000), Nuf2 (VDM4; 1:500), and PP1 (Santa-Cruz; sc-7482; 1:500) and probed using secondary antibodies labelled with infrared fluorescent dyes, which were imaged using an Odyssey (LiCOR) imager.

#### Kinetochore particle tracker

The Kinetochore-Particle-Tracker was developed in Python 3, using python’s image processing library scikit-image in Anaconda Environment and Jupyter Notebook. Data analysis was done in RStudio with the package ggplot. Figure panels were generated using matplotlib, ggplot and jupyter-notebook. Initial image pre-processing was done in ImageJ. To measure the kinetochore intensities in 3D images of time-lapse movies, the CENPB-dsRed signal was first detected to identify the location of kinetochores by applying an edge detector filter and a suitable threshold. Small particles were removed, and the holes were filled by performing morphological operations. Next, by burning the CENPB signal mask on the YFP-Astrin channel image, we extracted the mean particle intensities of YFP-Astrin. The cytoplasmic intensity was measured by creating a binary mask to segment the cell from the background and identifying a ring-shaped region as a proxy for the cytoplasm. The source code is available for download at Github: https://github.com/Draviam-lab/Kinetochore-Particle-Tracker.

#### CIVa database

CIVa database was developed in GitHub Pages using JavaScript, HTML and CSS. This database can be queried on the Gene Symbol or the Uniprot ID. The Git Page is available at: (https://draviam-lab.github.io/CIVa/). The source code is available at Github (https://github.com/Draviam-lab/CIVa).

### Quantification and statistical analysis

All experiments were repeated multiple times, as indicated in figure legends. Data were pooled and, if required, analyzed further in Microsoft Excel, and plotted in GraphPad Prism (v9.0; GraphPad Software, La Jolla, CA). Figure legends specify the n, errors, and the statistical test used. Data distributions were tested for normality using the D'Agostino-Pearson omnibus normality test and statistical differences among conditions were calculated using One-way ANOVA with DUNNET correction, Two-way ANOVA with Sidak correction, Chi-square test, Mann-Whitney U test (non-parametric) or paired t-test (parametric) in GraphPad Prism (v9.0; GraphPad Software, La Jolla, CA). Differences were considered significant if the p-value was <0.05 (∗), <0.01(∗∗), <0.001(∗∗∗), or < 0.0001 (∗∗∗∗), as indicated in each figure legend.

## Supplemental information

Document S1. Figures S1‒S11

Table S1. Includes predicted LoF variants in chromosome segregation genes and their allelic frequency, related to Figure 1
