# Supplementary material for: Search for chromosomal instability aiding variants reveal naturally occurring kinetochore gene variants that perturb chromosome segregation
Source: iScience. 2024 Jan 26;27(3):109007. doi: 10.1016/j.isci.2024.109007 (PMC10867425; doi:10.1016/j.isci.2024.109007)
Supplement: Document S1. Figures S1‒S11 [file mmc1.pdf]

iScience, Volume *27*

Supplemental information

Search for chromosomal instability aiding variants reveal naturally occurring kinetochore gene variants that perturb chromosome segregation

Asifa Islam, Janeth Catalina Manjarrez-González, Xinhong Song, Trupti Gore, and Viji M. Draviam

Supplemental Figures

Figure S1. Related to Figure 1.

A. Chromosomal Instability Variant (CIVa) analysis. Illustration of SKA3 gene highlighting its variant prevalence in COSMIC, gnomAD, Biomuta and ELGH databases. Images of lollipop graphs showing the positions and number of occurrences of different types of variations in the SKA3 gene, including potential LoF variant p.Q70Kfs*7 (marked in red). A black dot indicates a truncating mutation, a green dot indicates a missense mutation, and a purple dot indicates other mutation types. **B. Cancer mutational spectra.** Heat map of incidence of variations in selected genes in different tumour types in the COSMIC database. Box highlights genes with a low percentage of tumour samples showing somatic mutations.

Figure S2. Astrin variant p.Q1012* localises normally at the spindle but not at the kinetochores in the presence or absence of endogenous Astrin. Related to Figure 1.

A. Experimental regimen showing experiments with or without siRNA treatment wherein MG132 was used to arrest cells in metaphase before immunostaining or imaging. The qualitative analysis scheme describes ‘No’, ‘Low’, ‘Medium’ and ‘High’ crescent scores to distinguish the extent of Astrin crescents (in green) using a centromeric marker (in pink). **B.** Immunoblot showing Astrin depletion extent in cells treated as in A. **C.** Box plot showing Astrin localisation extent at kinetochores of Astrin siRNA treated cells expressing wild-type or variant as shown in **Fig 1B** (scored as high, medium, low and no crescents as in **A**). Symbols represent independent experiments. Two-way ANOVA with Sidak correction was performed for statistical significance. “****” represents $p<0.0001$. **D.** Cartoon showing N-terminally YFP-tagged Astrin wild-type, p.Q1012* variant and 4A and Δ70 mutant encoding cDNA design. Details of mutants are described previously[35]. **E.** Representative immunofluorescence images of Astrin wild type and p.Q1012*, 4A and Δ70 expressing cells treated as in A and probed for GFP and CREST. DNA was stained with DAPI. Cells were transfected with YFP-tagged Astrin (wild-type) or mutants and fixed for immunostaining following ~1 hour of MG132 treatment. Scale bars: 5 µm in uncropped images and 1 µm in insets. **F.** Box plot showing Astrin localisation extent at kinetochores of cells expressing Astrin wild-type, variant or mutants as shown in **E** (scored as high, medium, low and no crescents as in **A**). Symbols represent independent experiments. Two-way ANOVA with Sidak correction was performed for statistical significance. “****” represents $p<0.0001$.

Figure S3. SKA3 variant Q70Kfs*7 promotes a truncated SKA3 protein. Related to Figure 1.

A. Illustration showing the oligomerisation domain of SKA3 of the SKA complex, and the epitope region (156-177a.a of SKA3) recognised by the anti-SKA3 antibody, used in this study. **B.** Immunoblots showcasing a short fragment of SKA3 variant

expressed in cells transfected with plasmid vectors encoding N-terminal GFP-tagged SKA3 p.Q70Kfs*7 variant but not SKA3 WT, as indicated.

Figure S4 High frequency SKA3 (pR27*) does not disrupt chromosome congression. Related to Figure 2.

A. Table showing a set of variants in SKA3 gene reported in gnomAD allowing the assessment of variants that show increased prevalence in heterozygous compared to homozygous form. **B.** Representative deconvolved images of live mitotic cells coexpressing CENPB-DsRed (centromere marker) and N-terminal GFP-tagged SKA3 wild-type, p.Q70Kfs*7 or p.R27* variants as indicated (n = 18 for wild-type, 15 for p.Q70Kfs*7 or 15 for p.R27* expressing mitotic cells). **C.** Immunoblots showcasing short fragment of SKA3 variant expressed in cells transfected with plasmid vectors encoding N-terminal GFP-tagged SKA3 wild-type, p.Q70Kfs*7 or p.R27* variants as indicated. Tubulin was used as a loading marker.

Figure S5. Astrin variant and mutant designed to study Astrin p.LQfsTer21. Related to Figure 3.

A. Schematic of p.LQfsTer21 variant and p.L7* mutant. Red highlight show nucleotide alterations to mimic the variant. **B.** Cartoon showing N-terminal Astrin mutant encoding cDNA designs mimicking alternate (Kozak) start sites. CC1 and CC2 refer to coiled coil stretches predicted in Astrin dimers.

Figure S6. Astrin ∆274 localises normally at spindle and kinetochores when endogenous Astrin is depleted. Related to Figure 3.

A. Experimental regimen showing siRNA treatment (i) or plasmid DNA transfection alone (ii) times followed by MG132 treatment, fixation, immunostaining and imaging. **B.** Representative immunofluorescence images of cells expressing N-term YFP tagged Astrin wild type (WT), Δ151 and Δ274 mutants treated as in **A (ii)** and immunostained using an anti-GFP antibody (for YFP) and CREST antisera. DNA was stained with DAPI. Scale bars: 5 µm in uncropped images and 1 µm in insets. **C.** Box plot showing Astrin localisation extent at kinetochores (scored as high, medium, low and no crescents as in Fig 1S 2A). Symbols represent independent experiments. Two-way ANOVA with Sidak correction was performed for statistical significance. '*' and 'ns' represent '$p<0.05$' and 'not significant' respectively. **D.** Representative immunofluorescence images of YFP-tagged Astrin wild type (WT) and ∆274 expressing cells treated as in A (i) and immunostained using antibodies against GFP and SKAP and CREST antisera. DNA was stained with DAPI. Scale bars: 5 µm in uncropped images and 1 µm in insets. **E.** Box plot showing Astrin or SKAP localisation at kinetochores (scored as high, medium, low and no crescents as in Figure 1S 2A) in the absence of endogenous Astrin. Symbols represent independent experiments. Two-way ANOVA with Sidak correction was performed for statistical significance. * and ns represent $p<0.05$ and 'not significant' respectively.

Figure S7. Endogenous Astrin and SKAP localization is disrupted in Astrin p.Q1012* expressing cells. Related to Figure 4.

A. Experimental regimen showing plasmid DNA transfection and MG132 treatment to arrest cells in metaphase prior to immunostaining. **B.** Representative immunofluorescence images of Astrin wild type and p.Q1012* expressing cells treated as in A and probed for GFP, Astrin and CREST. DNA was stained with DAPI. Scale bars: 5 μm in uncropped images and 1 μm in insets. **C.** Violin plot showing the percentage of kinetochores where Astrin is localised as a crescent. Forty kinetochores were counted per cell. **D.** Representative immunofluorescence images of wild type and p.Q1012* expressing cells treated as in A and probed for GFP, SKAP and CREST. DNA was stained with DAPI. Scale bars: 5 μm in uncropped images and 1 μm in insets. **E.** Violin plot showing the percentage of kinetochores where SKAP localised as a crescent. Forty kinetochores were counted per cell.Dots represent independent cells, the solid line represents the median, dotted lines represent the quartiles and colours represent independent sets. Mann-Whitney U test was performed for statistical significance. '****' and '**' represent $p<0.0001$ and $p<0.01$, respectively.

Figure S8. Inter-kinetochore stretching is reduced in cells expressing Astrin p.Q1012*. Related to Figure 4.

A. Experimental regimen showing timepoints of plasmid transfection and MG132 treatment to arrest cells in metaphase prior to live-cell microscopy.
B. Representative time-lapse images of Astrin wild-type and p.Q1012* expressing cells treated as in A. Scale bars: 5 μm in uncropped images and 1 μm in insets. **C.** Cartoon showing inter-centromeric distance as a measurement of inter-kinetochore (KT) stretching. **D.** Scatter plot showing inter-KT distances in cells treated as in A measured for five pairs of KTs per cell over 10 minutes (1 frame/min). Colours represent different sets. 'n' is the number of cells. Error bars show mean with SD. Mann-Whitney U test was performed for statistical significance. '**' represents $p<0.001$. **E-F.** Change in inter-KT distances (normalised to least inter-KT distance) over time in cells treated as in A. "0" is the time point of least inter-KT distance. The shaded area represents a 95% confidence interval. 'nKT' is the number of kinetochores. (D-F) Data represent three independent experiments.

Figure S9. Kinetochore particle intensities show a steady reduction in Astrin p.Q1012* variant levels compared to wild-type. Related to Figure 4.

A. Automation routine for measuring YFP Astrin intensities at kinetochore particles. A particle mask was developed using CENPB-ds-Red (a centromere marker) image channel and then burned on the YFP-Astrin image channel. The detected kinetochore particles are then labelled and the mean intensity of Astrin at the particle site is calculated. The particles are coloured by their mean intensity values (Dark blue to light blue: high to low intensity). Scale bar: 5 μm **B.** Violin Plot showing the distribution of Astrin intensities at kinetochore (KT) particles in wild-type and p.Q1012* expressing cells. The box plot shows the mean kinetochore intensity is

less in p.Q1012* than in the wild-type. Mann-Whitney U test was performed for statistical significance. '****' represents $p < 0.0001$. n refers to the number of cells. **C.** Time-lapse analysis of Astrin intensities. Jitter is added to avoid overplotting. Each jitter point is the mean of the intensity ratio at that time point per cell for each condition.

Figure S10. Astrin p.Q1012* expression promotes congression failure and mitotic arrest. Related to Figure 4.

A. Violin plot showing the percentage of Astrin wild type and Astrin p.Q1012* expressing cells which successfully exited mitosis. Each dot represents an independent experiment. **B.** Violin plot showing the percentage of Astrin wild type and Astrin p.Q1012* expressing cells that successfully maintained chromosome congression. Each dot represents an independent experiment. The solid and dotted lines represent the median and quartiles, respectively. '*' and '**' represents $p<0.05$ and $p<0.01$, respectively.

Figure S11. A scalable framework for Chromosomal Instability Variant (CIVa) prediction and stratification. Related to Figure 4.

Illustration showing the prevalence of CIVa in chromosome segregation genes in the population, and stratification of CIVa (harmless *versus* harmful) based on their loss of kinetochore localisation and/or chromosome segregation function in homozygous (biallelic) or heterozygous (monoallelic) forms.

Figure S1

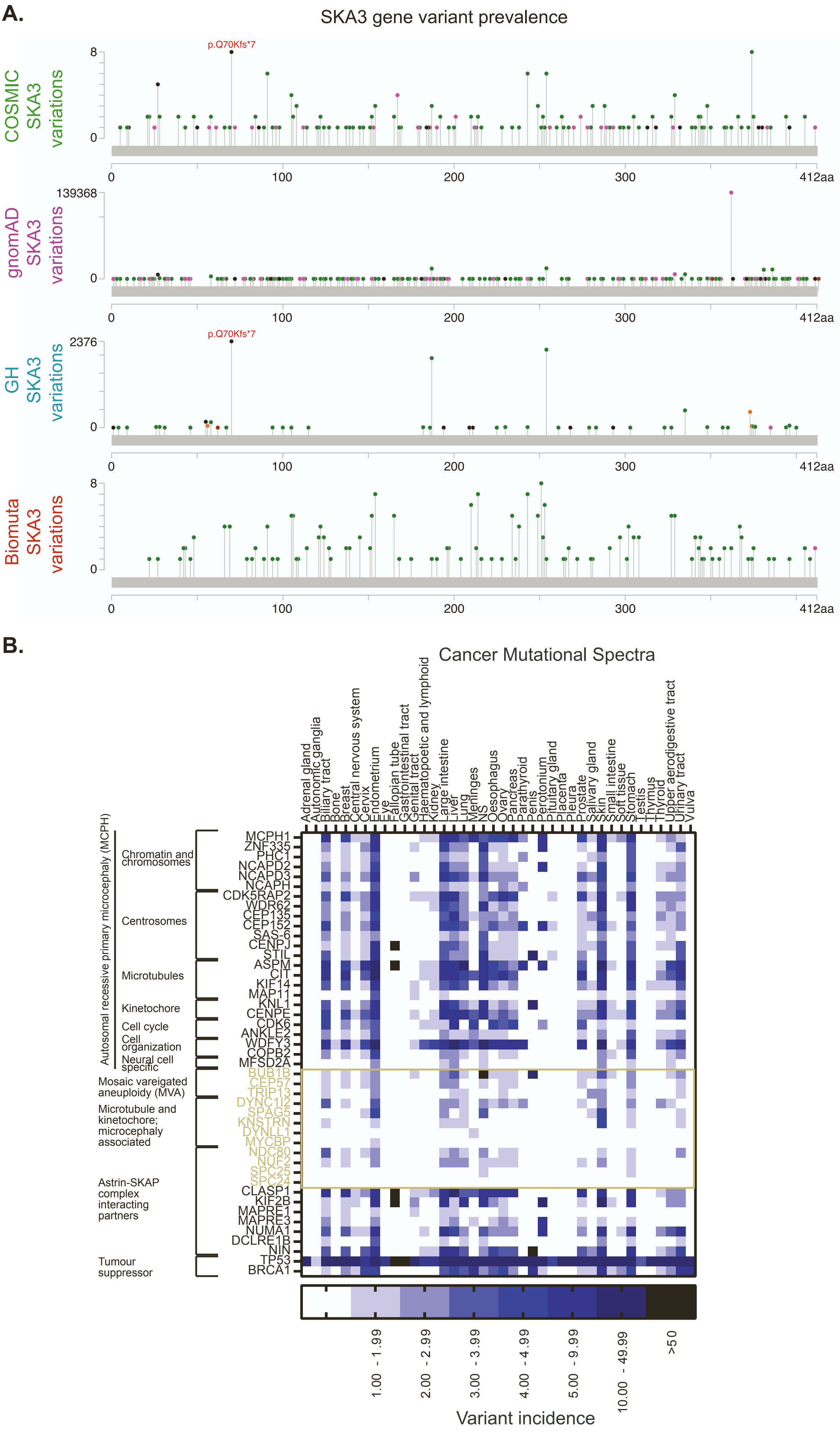
A.
SKA3 gene variant prevalence
COSMIC SKA3 variations
gnomAD SKA3 variations
GH SKA3 variations
Biomuta SKA3 variations
p.Q70Kfs*7
8
0
139368
2376
100
200
300
412aa
B.
Cancer Mutational Spectra
Adrenal gland
Autonomic ganglia
Biliary tract
Bone
Breast
Central nervous system
Cervix
Endometrium
Eye
Fallopian tube
Gastrointestinal tract
Genital tract
Haematopoetic and lymphoid
Kidney
Large intestine
Liver
Lung
Meninges
NS
Oesophagus
Ovary
Pancreas
Parathyroid
Penis
Peritonium
Pituitary gland
Placenta
Pleura
Prostate
Salivary gland
Skin
Small intestine
Soft tissue
Stomach
Testis
Thymus
Thyroid
Upper aerodigestive tract
Urinary tract
Vulva
Autosomal recessive primary microcephaly (MCPH)
Chromatin and chromosomes
Centrosomes
Microtubules
Kinetochore
Cell cycle
Cell organization
Neural cell specific
Mosaic vareigated aneuploidy (MVA)
Microtubule and kinetochore; microcephaly associated
Astrin-SKAP complex interacting partners
Tumour suppressor
MCPH1
ZNF335
PHC1
NCAPD2
NCAPD3
NCAPH
CDK5RAP2
WDR62
CEP135
CEP152
SAS-6
CENPJ
STIL
ASPM
CIT
KIF14
MAP11
KNL1
CENPE
CDK6
ANKLE2
WDFY3
COPB2
MFSD2A
BUB1B
CEP57
TRIP13
DYNC1I2
SPAG5
KNSTRN
DYNLL1
MYCBP
NDC80
NUF2
SPC25
SPC24
CLASP1
KIF2B
MAPRE1
MAPRE3
NUMA1
DCLRE1B
NIN
TP53
BRCA1
1.00 - 1.99
2.00 - 2.99
3.00 - 3.99
4.00 - 4.99
5.00 - 9.99
10.00 - 49.99
>50
Variant incidence

Figure S2

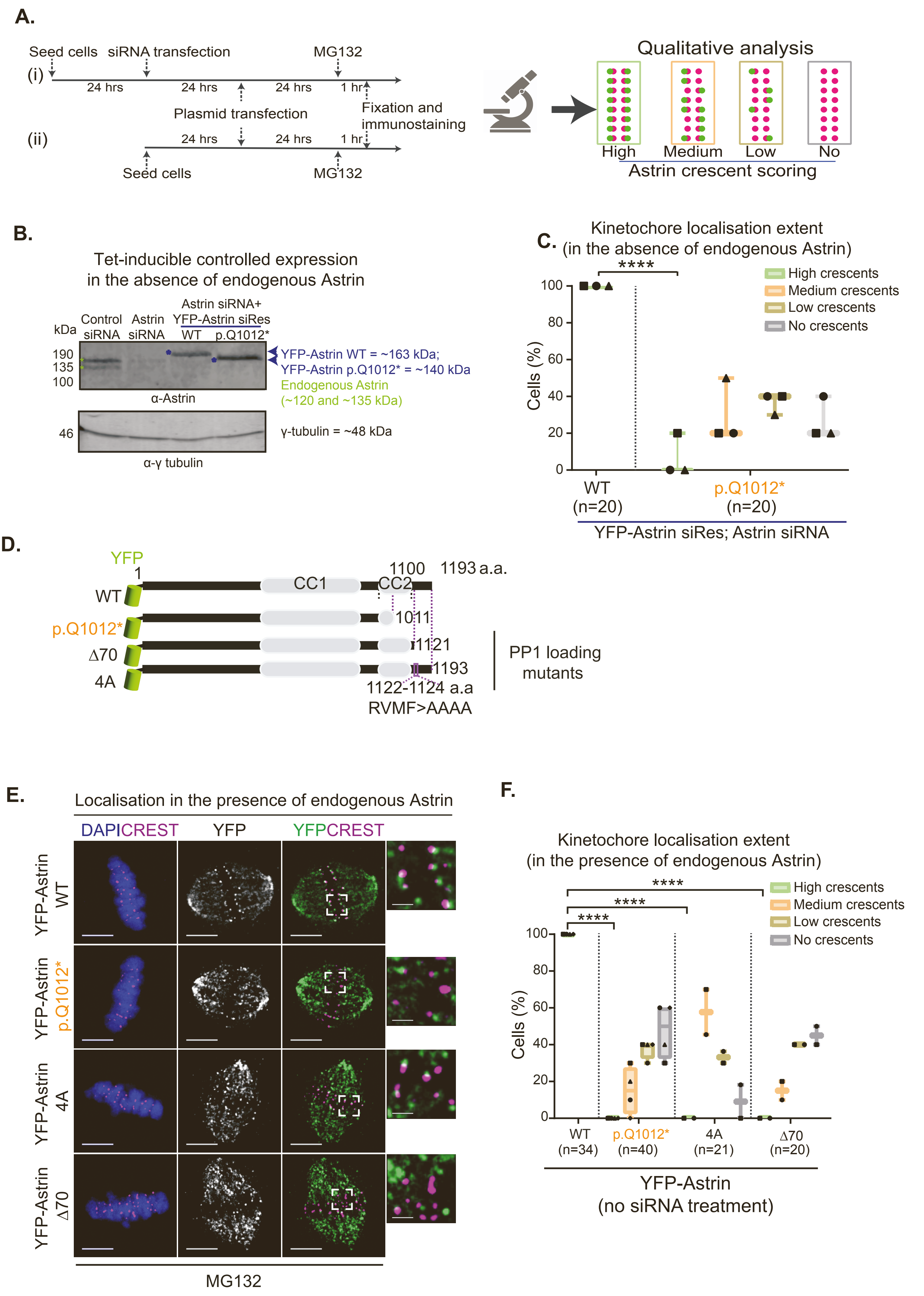

A.
Seed cells
siRNA transfection
MG132
(i)
24 hrs
24 hrs
24 hrs
1 hr
Plasmid transfection
Fixation and immunostaining
(ii)
24 hrs
24 hrs
1 hr
Seed cells
MG132
Qualitative analysis
High
Medium
Low
No
Astrin crescent scoring
B.
Tet-inducible controlled expression in the absence of endogenous Astrin
Astrin siRNA+
Control siRNA
Astrin siRNA
YFP-Astrin siRes
WT
p.Q1012*
kDa
190
135
100
α-Astrin
YFP-Astrin WT = ~163 kDa;
YFP-Astrin p.Q1012* = ~140 kDa
Endogenous Astrin (~120 and ~135 kDa)
46
α-γ tubulin
γ-tubulin = ~48 kDa
C.
Kinetochore localisation extent (in the absence of endogenous Astrin)
****
High crescents
Medium crescents
Low crescents
No crescents
Cells (%)
100
80
60
40
20
0
WT (n=20)
p.Q1012* (n=20)
YFP-Astrin siRes; Astrin siRNA
D.
YFP
1
1100
1193 a.a.
CC1
CC2
WT
p.Q1012*
1011
Δ70
1121
4A
1193
1122-1124 a.a
RVMF>AAAA
PP1 loading mutants
E.
Localisation in the presence of endogenous Astrin
DAPICREST
YFP
YFPCREST
YFP-Astrin WT
YFP-Astrin p.Q1012*
YFP-Astrin 4A
YFP-Astrin Δ70
MG132
F.
Kinetochore localisation extent (in the presence of endogenous Astrin)
****
****
****
High crescents
Medium crescents
Low crescents
No crescents
Cells (%)
100
80
60
40
20
0
WT (n=34)
p.Q1012* (n=40)
4A (n=21)
Δ70 (n=20)
YFP-Astrin (no siRNA treatment)

Figure S3

A.

SKA3 Wild-type and variant domains

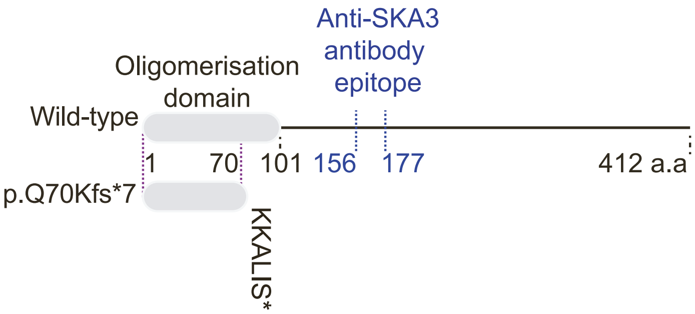

B.

SKA3 variant expression

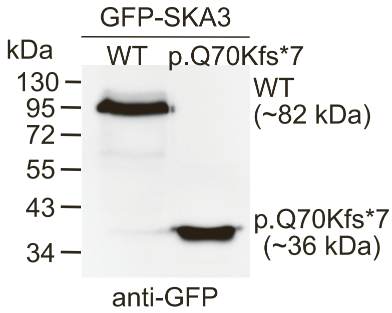

Figure S4

A.

Homozygous variant (relative prevalence)

**SKA3 (gnomAD)**	**Allele Count**	**Homozygous Number**	**Prevalence**
p.Arg27*	6805	0	Low
p.Ala28Val	1042	3	Moderate
p.Val58Ile	3966	75	High
p.Ser329Ser	7444	194	High
p.Asp335Glu	7432	194	High

B.

Chromosome congression analysis

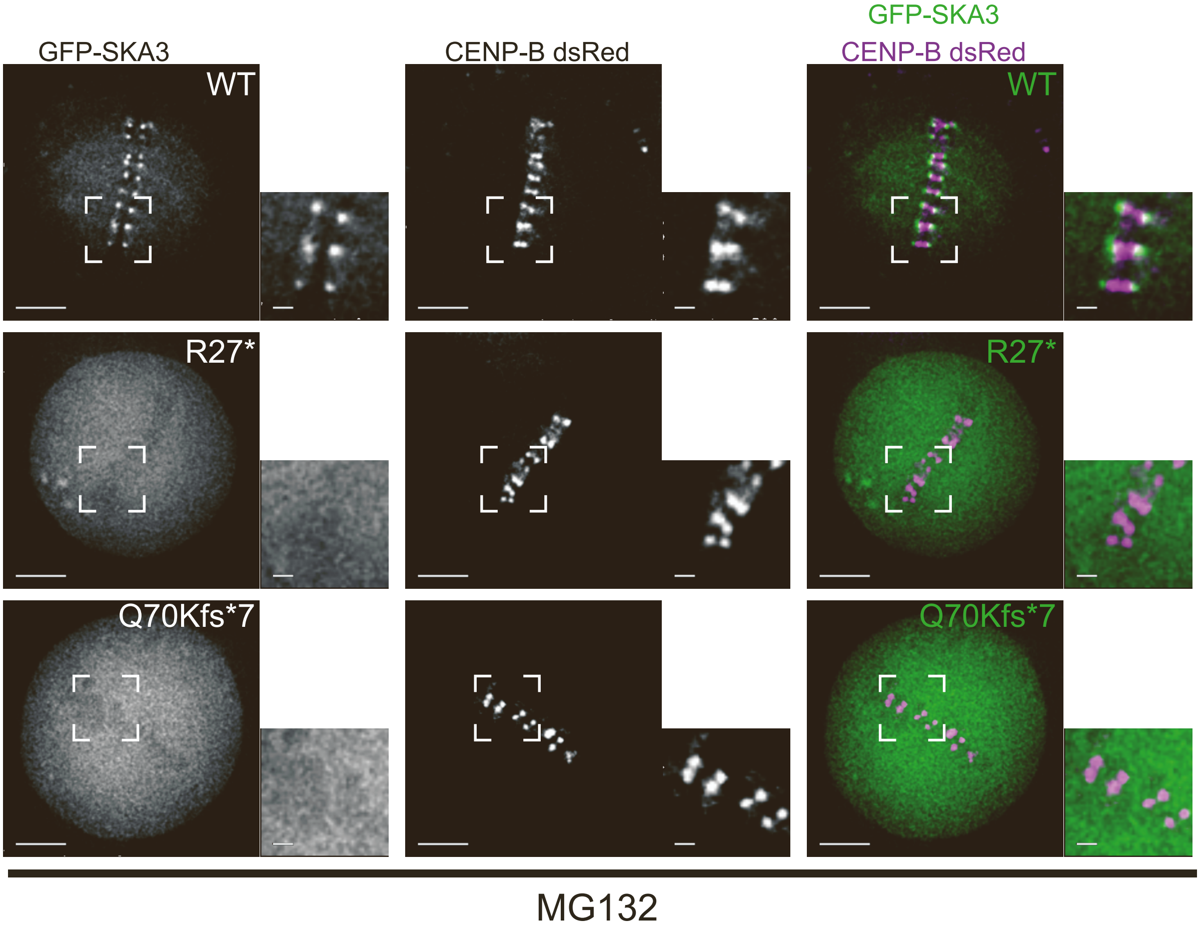

C.

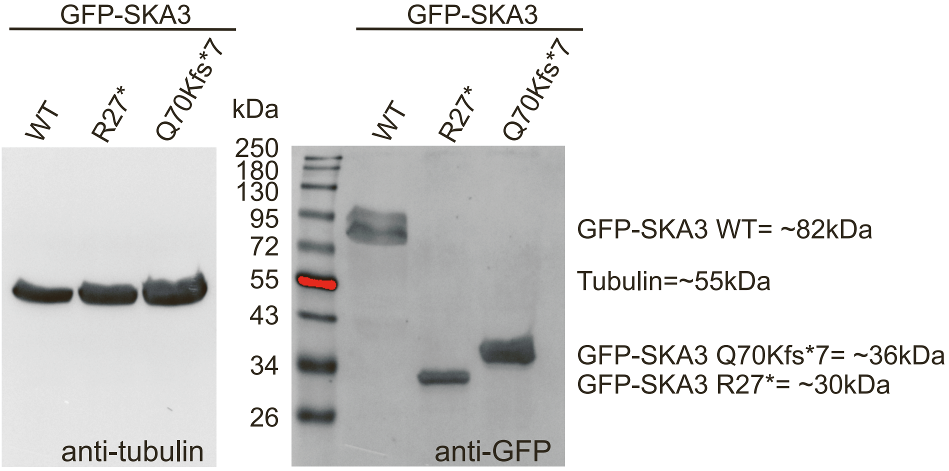

Figure S5

A.

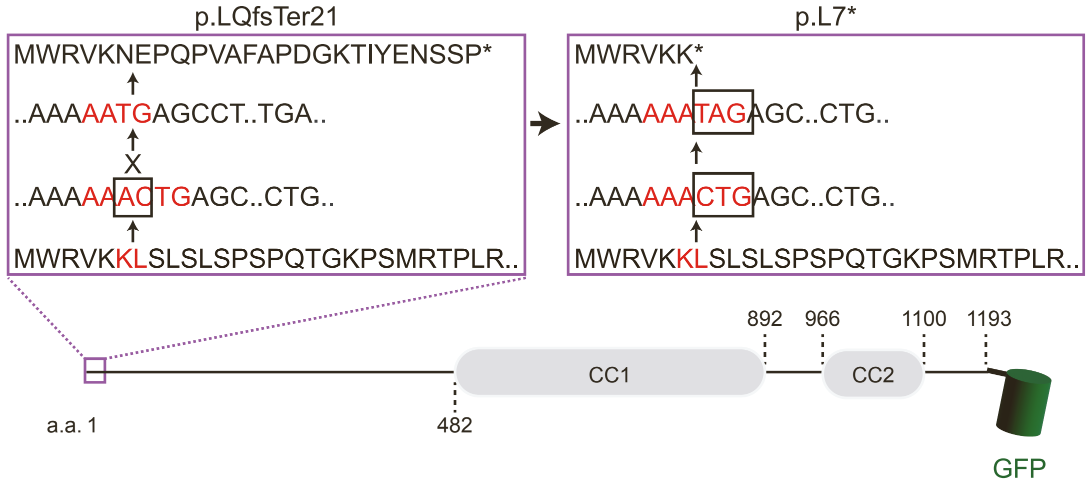
p.LQfsTer21
MWRVKNEPQPVAFAPDGKTIYENSSP*
..AAAAATGAGCCT..TGA..
X
..AAAAAACTGAGC..CTG..
MWRVKKLSLSLSPSPQTGKPSMRTPLR..
p.L7*
MWRVKK*
..AAAAAATAGAGC..CTG..
..AAAAAACTGAGC..CTG..
MWRVKKLSLSLSPSPQTGKPSMRTPLR..
892
966
1100
1193
CC1
CC2
a.a. 1
482
GFP

B.

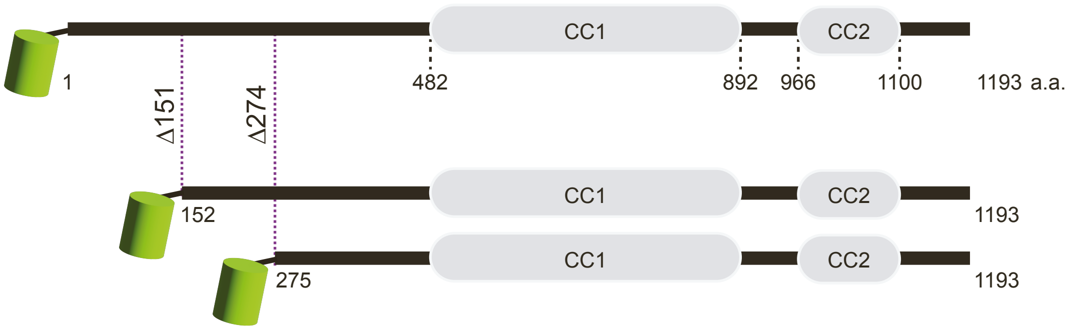
CC1
CC2
1
482
892
966
1100
1193 a.a.
Δ151
Δ274
152
1193
275
1193

Figure S6

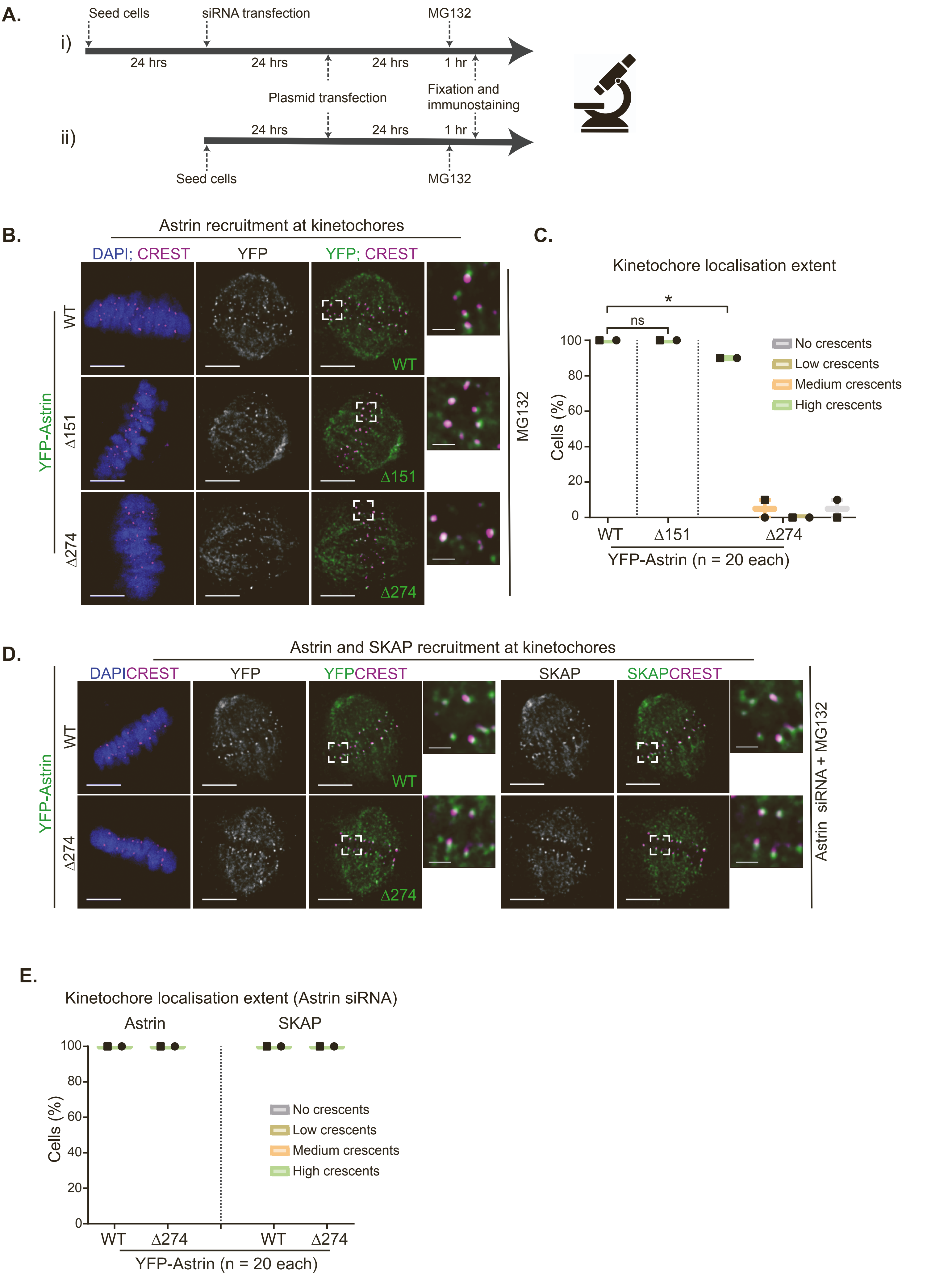

A.
i)
Seed cells
siRNA transfection
MG132
24 hrs
24 hrs
24 hrs
1 hr
Plasmid transfection
Fixation and immunostaining
ii)
24 hrs
24 hrs
1 hr
Seed cells
MG132
B.
Astrin recruitment at kinetochores
DAPI; CREST
YFP
YFP; CREST
YFP-Astrin
WT
Δ151
Δ274
MG132
C.
Kinetochore localisation extent
*
ns
Cells (%)
100
80
60
40
20
0
No crescents
Low crescents
Medium crescents
High crescents
WT
Δ151
Δ274
YFP-Astrin (n = 20 each)
D.
Astrin and SKAP recruitment at kinetochores
DAPICREST
YFP
YFPCREST
SKAP
SKAPCREST
YFP-Astrin
WT
Δ274
Astrin siRNA + MG132
E.
Kinetochore localisation extent (Astrin siRNA)
Astrin
SKAP
Cells (%)
100
80
60
40
20
0
No crescents
Low crescents
Medium crescents
High crescents
WT
Δ274
WT
Δ274
YFP-Astrin (n = 20 each)

Figure S7

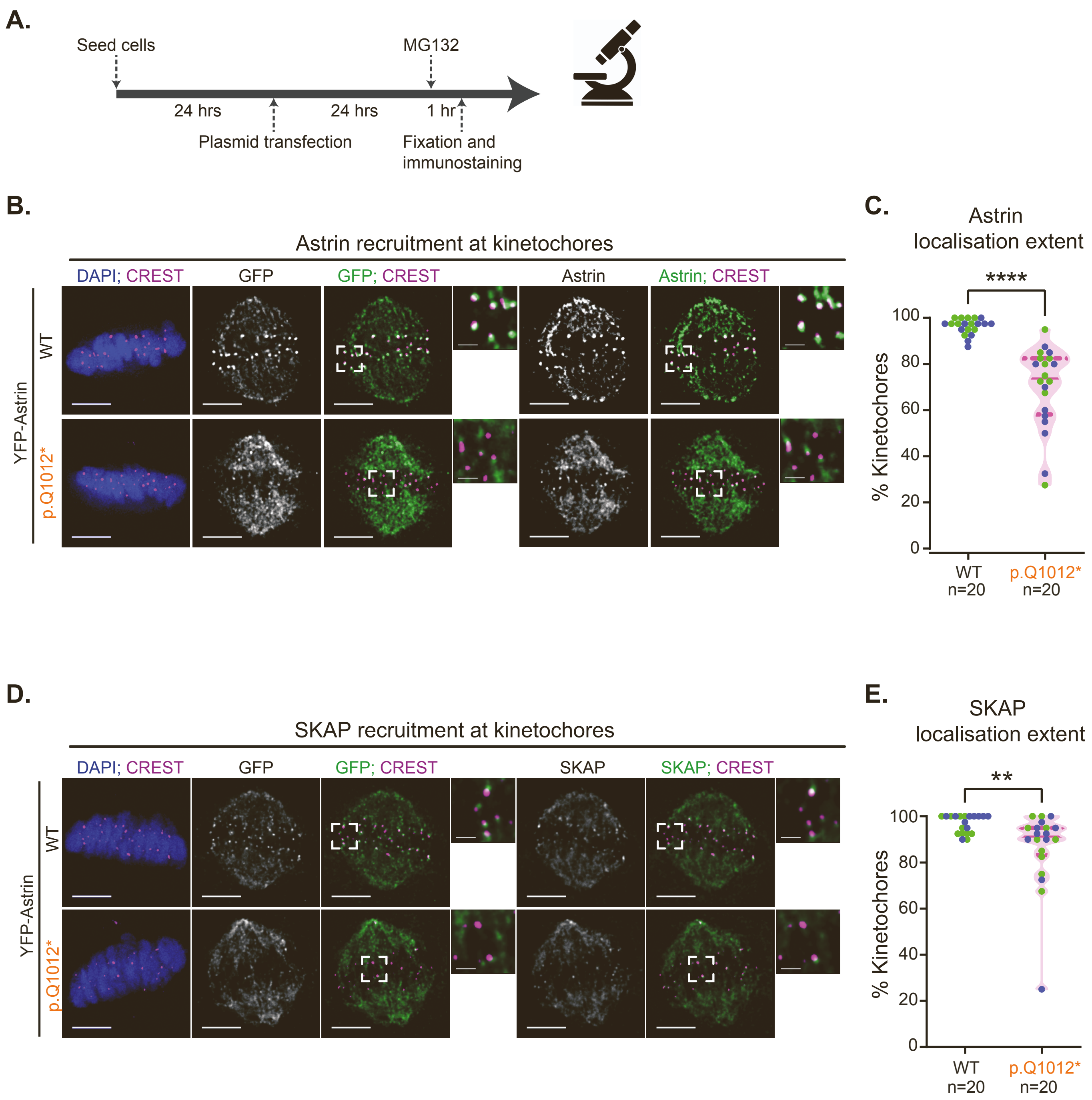
A.
Seed cells
MG132
24 hrs
24 hrs
1 hr
Plasmid transfection
Fixation and immunostaining
B.
Astrin recruitment at kinetochores
DAPI; CREST
GFP
GFP; CREST
Astrin
Astrin; CREST
YFP-Astrin
WT
p.Q1012*
C.
Astrin localisation extent
****
% Kinetochores
0
20
40
60
80
100
WT n=20
p.Q1012* n=20
D.
SKAP recruitment at kinetochores
DAPI; CREST
GFP
GFP; CREST
SKAP
SKAP; CREST
YFP-Astrin
WT
p.Q1012*
E.
SKAP localisation extent
**
% Kinetochores
0
20
40
60
80
100
WT n=20
p.Q1012* n=20

Figure S8

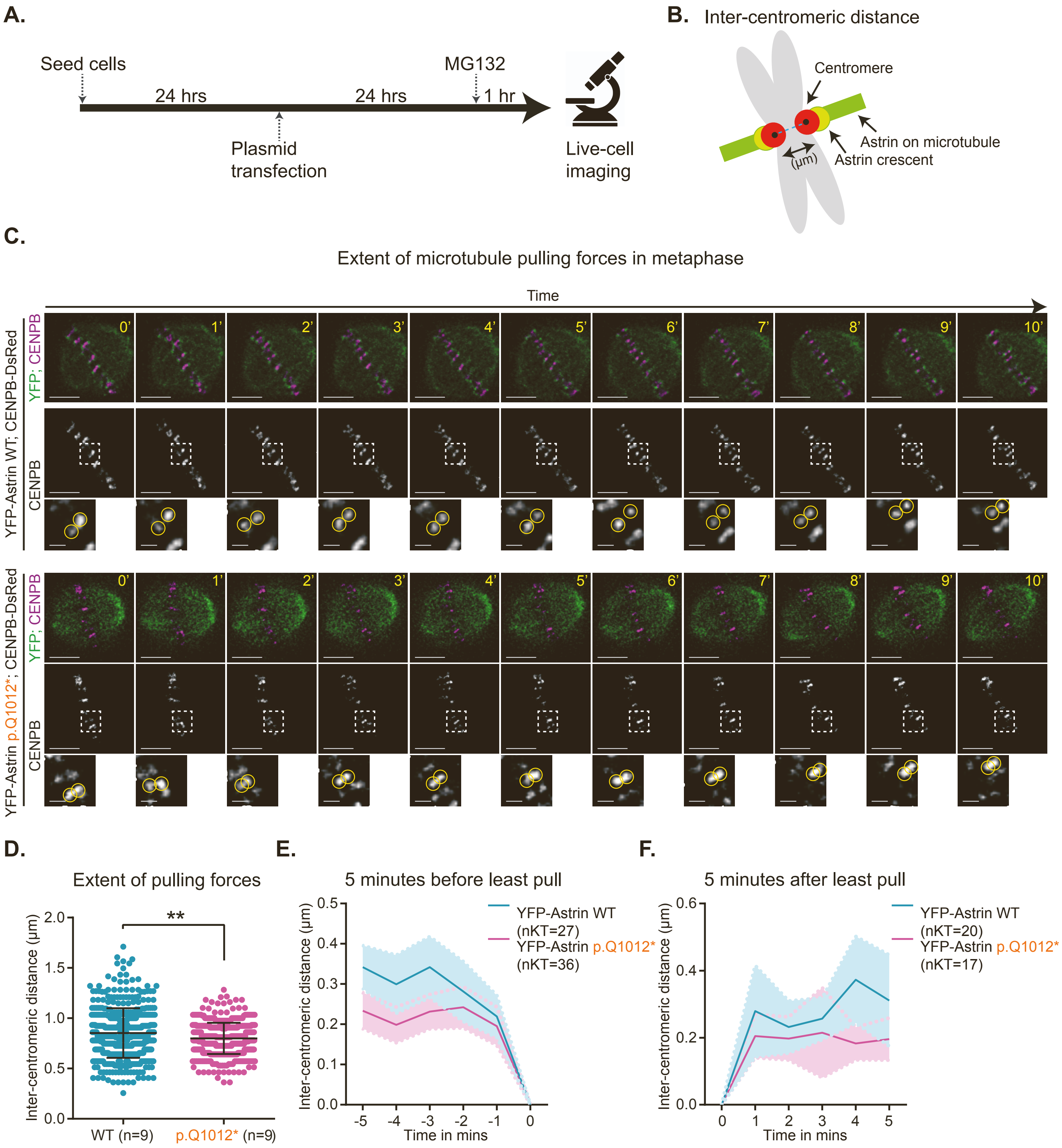
A.
Seed cells
24 hrs
Plasmid
transfection
24 hrs
MG132
1 hr
Live-cell
imaging
B.
Inter-centromeric distance
Centromere
Astrin on microtubule
Astrin crescent
(μm)
C.
Extent of microtubule pulling forces in metaphase
Time
YFP-Astrin WT; CENPB-DsRed
YFP; CENPB
CENPB
YFP-Astrin p.Q1012*; CENPB-DsRed
0'
1'
2'
3'
4'
5'
6'
7'
8'
9'
10'
D.
Extent of pulling forces
Inter-centromeric distance (μm)
**
WT (n=9)
p.Q1012* (n=9)
E.
5 minutes before least pull
Inter-centromeric distance (μm)
Time in mins
YFP-Astrin WT
(nKT=27)
YFP-Astrin p.Q1012*
(nKT=36)
F.
5 minutes after least pull
Inter-centromeric distance (μm)
Time in mins
YFP-Astrin WT
(nKT=20)
YFP-Astrin p.Q1012*
(nKT=17)

Figure S9

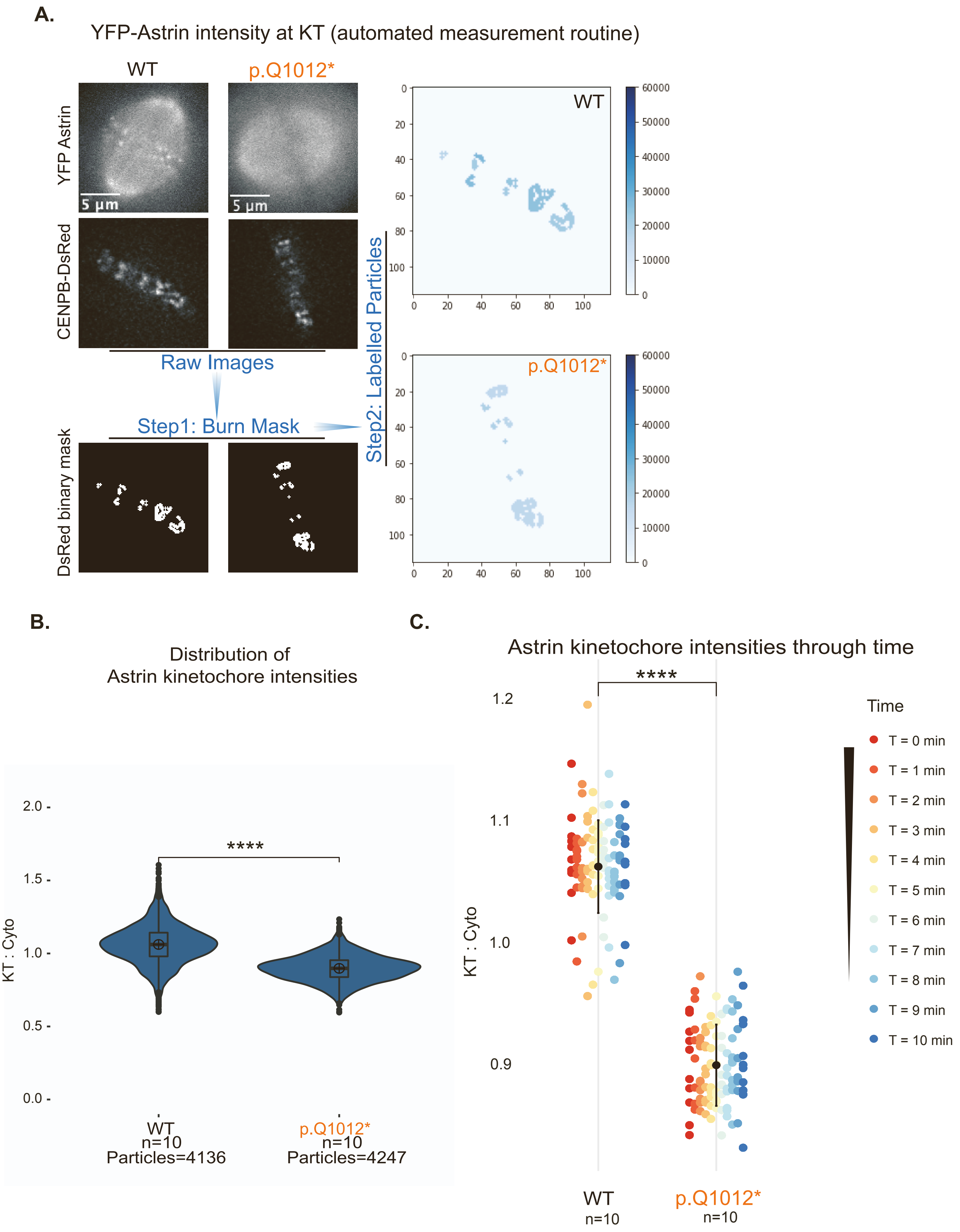
A.
YFP-Astrin intensity at KT (automated measurement routine)
WT
p.Q1012*
YFP Astrin
CENPB-DsRed
DsRed binary mask
5 µm
5 µm
Raw Images
Step1: Burn Mask
Step2: Labelled Particles
WT
p.Q1012*
0
20
40
60
80
100
60000
50000
40000
30000
20000
10000
0
B.
Distribution of
Astrin kinetochore intensities
KT : Cyto
2.0
1.5
1.0
0.5
0.0
****
WT
n=10
Particles=4136
p.Q1012*
n=10
Particles=4247
C.
Astrin kinetochore intensities through time
KT : Cyto
1.2
1.1
1.0
0.9
****
WT
n=10
p.Q1012*
n=10
Time
T = 0 min
T = 1 min
T = 2 min
T = 3 min
T = 4 min
T = 5 min
T = 6 min
T = 7 min
T = 8 min
T = 9 min
T = 10 min

Figure S10

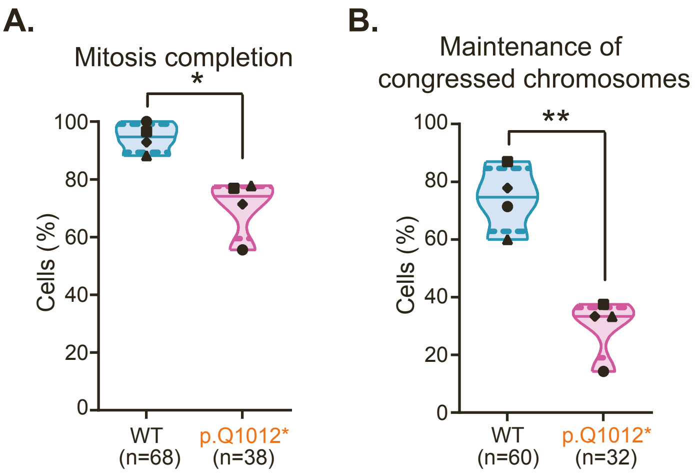
A.
Mitosis completion
*
Cells (%)
100
80
60
40
20
0
WT
(n=68)
p.Q1012*
(n=38)
B.
Maintenance of
congressed chromosomes
**
Cells (%)
100
80
60
40
20
0
WT
(n=60)
p.Q1012*
(n=32)

Chromosomal Instability aiding Variant (CIVa) prediction and ranking

CIVa database	Prevelance (Het/Hom)	Kinetochore localisation	Functional consequence	CIVa stratification
Astrin p.Q1012*	2/0	Failure	Misalignment Missegregation	Very Harmful
SKA3 p.Q70Kfs7*	1908/2	Failure	Normal alignment	Harmful (biallelic) Harmless (monoallelic)
SKA3 p.R27*	6805/0	Failure	Normal alignment	Harmless (monoallelic)
Astrin p.L7Qfs21*	431/6	Normal (short isoforms)	Normal alignment	Harmless

Increasing severity
